# Peripheral rotavirus-specific T-cell responses following monovalent oral rotavirus vaccine in infants

**DOI:** 10.1038/s41541-026-01405-z

**Published:** 2026-03-05

**Authors:** Alexander R. Nicols, Yesun Lee, Zion Congrave-Wilson, Minjun Kim, Wesley A. Cheng, Jaycee Jumarang, Jocelyn Navarro, Rafaella Navarro, Yamile Rodriguez-Angeles, David Durand, Zackary W. Taylor, Ruth G. De León, Theresa J. Ochoa, Alessandro Sette, Ricardo da Silva Antunes, Pia S. Pannaraj

**Affiliations:** 1 Division of Infectious Diseases, Department of Pediatrics, University of California, San Diego, La Jolla, CA USA; 2 David Geffen School of Medicine, University of California, Los Angeles, Los Angeles, CA USA; 3 Instituto de Medicina Tropical Alexander von Humboldt, Universidad Peruana Cayetano Heredia, Lima, Peru; 4 Laboratorios de Investigación y Desarrollo (LID), Universidad Peruana Cayetano Heredia, Lima, Peru; 5 Division of Pediatric Infectious Diseases, Kaiser Permanente, Los Angeles, CA USA; 6 Department of Sexual and Reproductive Health, Gorgas Memorial Institute for Health Studies, Panama City, Panama; 7 Center for Vaccine Innovation, La Jolla Institute for Immunology (LJI), La Jolla, CA USA; 8 Department of Medicine, Division of Infectious Diseases and Global Public Health, University of California, San Diego, La Jolla, CA USA; 9 Division of Infectious Diseases, Rady Children’s Hospital, La Jolla, CA USA

**Keywords:** Immunology, Microbiology

## Abstract

Despite evidence of varying vaccine effectiveness, T cell responses to rotavirus (RV) vaccines remain incompletely studied. To address this research gap, RV-specific T cells in the blood of infants pre- and post-monovalent RV vaccination (RV1) were analyzed for memory recall and functionality using RV-specific peptide pool stimulation. We find that RV vaccine elicits heterogenous responses with respect to cellular and humoral immunity. T cell responses to RV vaccine are detectable in the periphery, though poorly functional. Vaccination induces Th2-biased conventional effector memory and central memory CD4 + T cells, as suggested by chemokine receptor profiles, though the response wanes by 8 months post vaccination. The presence of preexisting immunity results in no significant increase in either RV-specific IgA or T cells after vaccination. Our data provides the first in-depth assessment of RV-specific T cell responses induced by vaccine, demonstrating patterns of negative and positive association with response that may play a role in protection against rotavirus disease.

## Introduction

Rotavirus (RV) is one of the leading causes of life-threatening diarrhea among young children^1,2^. Prior to the introduction of RV vaccines, approximately 600,000 deaths per year were attributed to RV diarrhea worldwide^2–4^. The majority of deaths occur in low-to-middle income countries (LMICs) such as India, Pakistan and areas of Sub-Saharan Africa^2–4^. Since the introduction of two orally administered RV vaccines in the mid-2000s, the number of annual deaths attributed to RV diarrheal disease has decreased by ~75%^3,5^. However, there remains significant disease burden in LMICs due to inadequate vaccination coverage and lower vaccine effectiveness (VE) in these populations^5–12^. Despite high VE ( ≥ 95%) in high income countries, several studies have shown significantly lower VEs in LMICs, with 12 month follow up efficacies as low as 44%^6,7,13–15^. This represents a significant effectiveness gap in areas of the world with the greatest need for protection.

The most widely purported surrogate of protection is a serum RV-specific IgA fold change after vaccination^16–21^. However, serum seroconversion alone is a suboptimal metric of vaccine response given the tendency for heterogeneity in peripheral immune responses^16,22,23^. Notably, the cellular immune response to RV vaccines remains poorly studied, with current knowledge of RV-specific T cells coming primarily from challenge studies in murine models and observational studies of natural infection in humans^22,24–27^. Work in immunodeficient mouse models has demonstrated that CD8 + T cells are required for infection clearance^24,25,27^. Complementary work in humans has revealed consistent patterns of limited T cell memory recall and poor functional responses of CD4+ and CD8 + T cells in the blood, typically defined by monofunctional expression of IFNγ^22,23,26,28–31^. RV vaccine driven antigen-specific T cell responses are seldom reported, but one study has revealed similar patterns to those observed in an infection setting^32^. Given the lack of substantial vaccine T cell response data, there is a critical need for a broad characterization of antigen-specific T cell memory to RV vaccination in infants. To address this, we studied T cell and antibody responses in infants from three socioeconomic settings: Panama, Peru and the United States.

## Results

### Participants and immune response characteristics

Between February 2018 and January 2023, 303 healthy infants were enrolled into the study prior to their first RV vaccine and provided pre- and post-RV vaccination blood samples from three sites (Los Angeles, California, U.S., Panama City, Panama, and Lima, Peru). Only those participants vaccinated with the oral RV1 (Rotarix®, GlaxoSmithKline Biologicals) regimen were included in the analyses. RV1 doses were given at 2 and 4 months of age according to the recommended schedule of each country. The median age (days of life) at vaccine administration was 63 at dose 1 (IQR, 61—70.5) and 127 at dose 2 (IQR, 123—143). Peripheral blood samples were obtained pre- and post-vaccine at 2 and 6 months of life (2 months post dose 2), respectively. Samples from 60 eligible participants were selected for analysis based on pre- and post- RV vaccine sample availability at the U.S.-based laboratory at the time of analysis and sufficient cell counts >3 million cells per vial for the assay’s requirements. Of those, 39 infants had samples at both pre-and post-vaccination time points that passed quality control measures (see methods) and were included in the final T cell analysis. Ten (25.6%) were Panamanian, 21 (53.8%) Peruvian and 8 (20.5%) US. Most (92.3%) participants were of Hispanic ethnicity.

T cell response status was determined by a ≥ 2-fold change in RV-specific CD4+ and CD8 + T cells between the pre- and post-vaccine timepoints. However, only CD4+ responses changed significantly between the vaccine timepoints and were thus the basis for T cell response calling. Despite lacking statistical significance, T cell responsiveness aligned with IgA-based response classification (median IgA fold change = 5.1 for T cell responders; IQR = 1.3–11.4 vs 1.5 for non-responders; IQR = 0.8–4.3, p = 0.1, Table 1). Serological vaccine response status was defined by a ≥ 3-fold change in serum RV-specific IgA measured by enzyme-linked immunosorbent assay (ELISA). Based on this, 16 participants were classified as antibody responders (median FC = 8.88, p < 0.0001), whilst 23 were non-responders (median FC = 1.13, Supplemental Fig. 1A, B). There was no significant difference in infant age at the administration of either dose between T cell response groups (Table 1). Country of origin, sex, race and ethnicity were not significantly associated with RV-specific T cell response status (Table 1).

**Table 1:** Cohort characteristics and demographics

	T-cell responder (≥2-fold change)	T-cell non-responder (≥2-fold change)	Significance
*n* (%)	13 (33.3%)	26 (66.6%)	
Country			
Panama	5 (50.0%)	5 (50.0%)	NS
Peru	7 (33.3%)	14 (66.7%)	
US	2 (25.0%)	6 (75.0%)	
Race			
White	5 (50.0%)	5 (50.0%)	NS
African American	0	2 (100.0%)	
Mixed	8 (30.8%)	18 (69.2%)	
American Indian	1 (100%)	0	
Ethnicity			
Hispanic	13 (36.1%)	23 (63.9%)	NS
Non-hispanic	1 (33.3%)	2 (66.6%)	
Sex			
Female	6 (33.3%)	12 (66.6%)	NS
Male	8 (38.1%)	13 (61.9%)	
Age at vaccination (days)			
Dose 1 median (IQR)	63 (61–102)	64 (62–70)	NS
Dose 2 median (IQR)	127.5 (124–156)	127 (124–136)	NS
Median RV-specific IgA fold change (IQR)	5.1 (1.3–11.4)	1.5 (0.8–4.3)	0.10
RV-specific IgA responder			
Yes	8 (50.0%)	8 (50.0%)	NS
No	6 (26.1%)	17 (73.9%)	

*RV* rotavirus, *Ig* immunoglobulin, *IQR* interquartile range, *NS* not significant. Comparisons between sex in T cell responders and non-responders were made by a two-sided Fishers exact test. Comparisons between country, race, and ethnicity were made using a Chi-squared test. All other comparisons were made using Mann–Whitney U tests.

### RV vaccine elicits conventional and regulatory CD4 + T cell responses in the blood with strong effector and central memory phenotypes but poor functionality

**Fig. 1:**
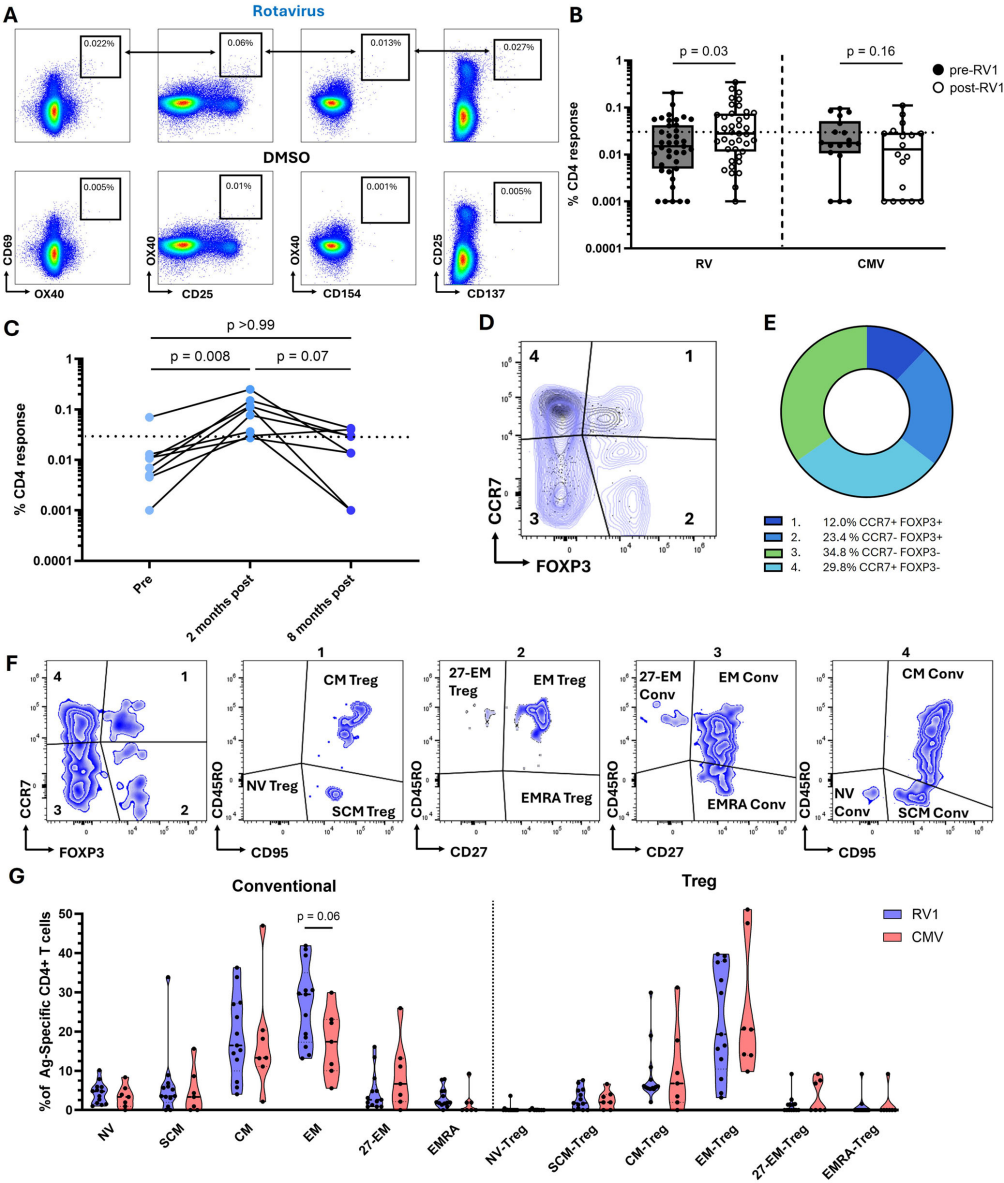
CD4 + T cell responses to RV vaccine in infants. **A** Representative gating strategy for AIM + CD4 + T cell response calling by using Boolean OR gating. Events were pre-gated as live, single CD3 + CD4 + T cells. **B** Paired analysis of pre- vs post-RV vaccine magnitudes of RV- (n = 39) and CMV-specific (n = 18) CD4 + T cells, expressed as a % of total CD4 + T cells. Data are shown with median, quartiles and minimum/maximum values. **C** Matched paired comparisons of RV-specific CD4 + T cell magnitude pre vaccination, 2 months post (6 months of life) and 8 months post dose 2 (12 months of life), n = 8. **D** Representative gating strategy for splitting Ag-specific CD4 + T cells into 4 core subsets by CCR7 and FOXP3. **E** Visual representation of the proportional composition of RV-specific CD4 + T cells in RV-vaccine T cell responders post vaccine (n = 13). **F** Representative gating strategy to determine extended memory subsets of conventional and regulatory Ag-specific CD4 + T cells. **G** Comparison (unpaired) of T cell memory phenotypes between in RV-vaccine T cell responders (n = 13) and CMV-specific (n = 7) conventional and regulatory CD4 + T cells post vaccine. Data are shown with the median and quartiles. Comparisons across multiple paired or unpaired groups were made by Friedman’s or Kruskal-Wallis with Dunns post hoc test, respectively. Matched pairwise comparisons were made by Wilcoxon matched signed rank tests. Comparisons between unpaired RV and CMV phenotypes were made by Mann-Whitney tests p = <0.05. Significant values or those nearing significance (p < 0.1) are displayed. Phenotyping magnitudes are plotted as the percentage of the total Ag-specific CD4 + T cell response. Data points represent individual infants. Horizontal dotted lines represent the limit of sensitivity (LOS) as determined by the median + 2 x standard deviation of a synthetic non-specific myelin oligodendrocyte glycoprotein (MOG) peptide response (**B**, **C** = 0.031). AIM activation induced marker, PHA phytohemagglutinin-L, RV rotavirus, CMV cytomegalovirus, NV naive, SCM stem cell memory, EM effector memory, EMRA effector memory cells re-expressing CD45RA.

RV-specific CD4 + T cells in peripheral blood increased significantly across the cohort from pre- to post-RV vaccine, when evaluated by both magnitude (p = 0.03, Fig. 1A, B) and stimulation index (p = 0.03, Supplementary Fig. 2A, B). To compare responses to another common infant antigen, cytomegalovirus (CMV)-specific T cells were also assayed in the blood of RV-vaccinated infants in this cohort. Given the lack of consensus on how T cell response to vaccination is defined in the literature, we categorized participants as T cell RV vaccine responders or non-responders based on a ≥ 2-fold change in AIM + RV-specific CD4 + T cell magnitude after vaccination. Based on this, 13/39 (33.3%) participants were identified as RV vaccine T cell responders. Unlike the increase in RV-specific CD4 + T cells induced by RV vaccine, the magnitude of CMV-specific CD4 + T cells did not change (Fig. 1B). 12/39 participants were found to be CMV-T cell responders based on magnitudes above the LOS and stimulation indexes ≥2 at either timepoint, of which 4/12 (33.3%) were also RV-T cell vaccine responders. Next, we investigated the long-term persistence of RV-specific CD4 + T cell magnitudes in blood samples taken 8 months (median 241 days) post second dose of RV vaccine (12 months of life). We observed waning of the RV-specific CD4 + T cell response in the peripheral blood of infants (Fig. 1C). Importantly, there was evidence of individual variation with respect to the degree and severity of waning (Fig. 1C). Seven of 8 (87.5%) participants were below the LOS pre-vaccine, with most (7/8[87.5%]) responding above the LOS 2 months after vaccination. Waning varied among participants with 5/8 (62.5%) dropping back below the LOS 8 months after vaccine (Fig. 1C). Both RV- and CMV-specific CD4 T cells were poorly functional in these infants (Supplemental Fig. 3).

To analyze RV-specific CD4 + T cells phenotypically, post-vaccine CD4 + T cells of the 13 T cell responders were split into conventional or regulatory subgroups based on CCR7 and FOXP3 expression (Fig. 1D). RV-specific CD4 + T cell phenotypes were compared to CMV-specific phenotypes of those participants with CMV responses above the LOS. The majority of the CD4 + T cell response to RV vaccine displayed a conventional phenotype with 34.8% being CCR7− FOXP3− and 29.8% CCR7 + FOXP3− (Fig. 1E). The bulk of the regulatory T cell response was CCR7− (23.4%) whilst CCR7+ regulatory cells made up the smallest proportion of the RV-specific CD4 + T cell response (12.0%) (Fig. 1E). To capture the full breadth of RV-specific CD4 + T cell phenotypes post vaccine, the 4 subgroups were further characterized based on the expression of additional memory phenotyping markers including CD45RO, CD27 and CD95 (Fig. 1F). This extended analysis revealed that the majority of RV-specific FOXP3+ regulatory CD4 + T cells post vaccine were of an effector memory (CCR7− CD45RO + CD27 + EM) or central memory (CCR7 + CD45RO + CD95 + CM) phenotype, similar to CMV-specific responses (Fig. 1G). Conventional RV-specific CD4 + T cells were also mostly of the EM and CM type, with stem cell memory (CCR7 + CD45RO− CD95 + SCM) and CD27- effector memory (CCR7− CD45RO + CD27−) making up smaller proportions of the overall response. The post vaccine response of conventional RV-specific CD4s were similar in phenotype to CMV-specific cells (Fig. 1G).

### Rotavirus specific conventional CD4 + T cells post RV vaccine are biased toward a Th2 chemokine receptor profile

**Fig. 2:**
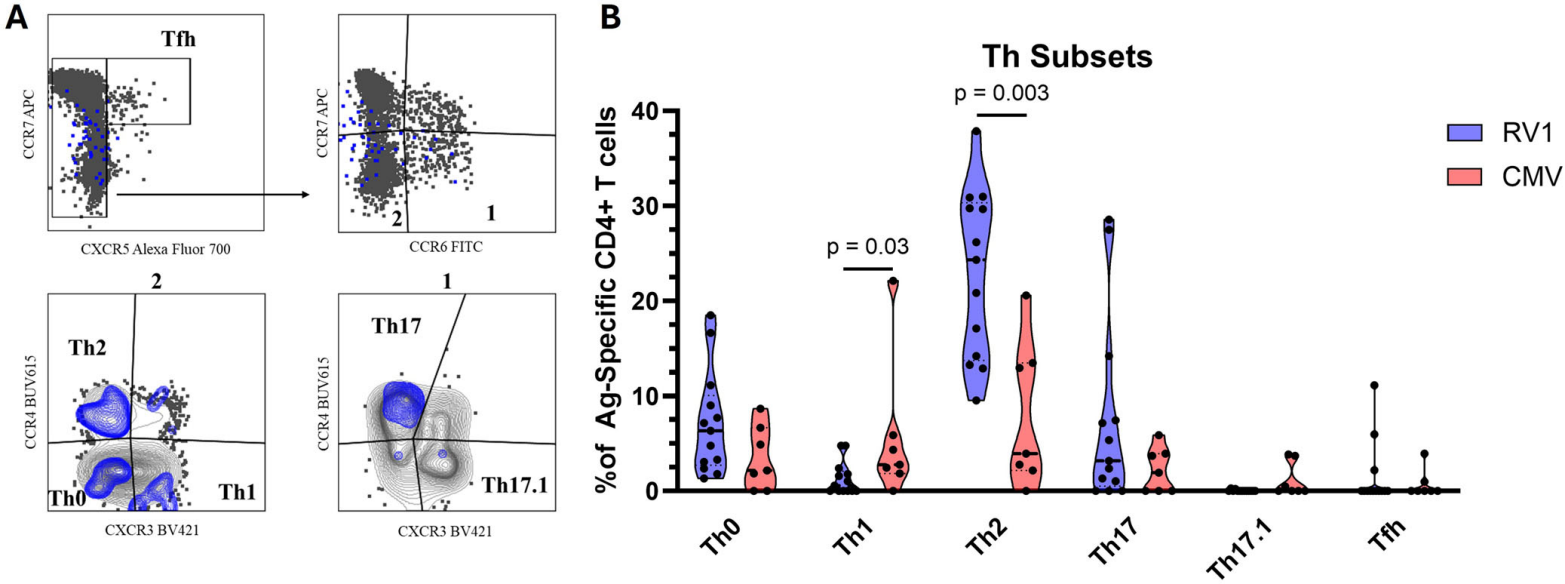
Chemokine receptor analysis and Th polarization among RV- and CMV-specific CD4 + T cells. **A** Representative flow cytometry plots showing the gating strategy for chemokine receptor analysis. Bulk CD4 + T cells are plotted in grey with Ag-specific events overlayed in blue. **B** Unpaired comparison of T cell chemokine receptor phenotypes between RV- (n = 13) and CMV-specific (n = 7) FOXP3− CD4 + T cells post vaccine in participants with AIM+ responses above the LOS with stimulation indexes ≥2. Data are shown with the median and quartiles. Data points represent individual infants. Magnitudes are plotted as the percentage of the total Ag-specific CD4 + T cell response. Comparisons between RV and CMV chemokine receptors phenotypes were made by Mann-Whitney tests p = <0.05. Significant p values are displayed. Ag antigen, RV rotavirus, CMV cytomegalovirus, Th T-helper cell.

Having found that RV-vaccine induces strong memory phenotypes, we next sought to compare the chemokine receptor profiles of conventional RV- and CMV-specific FOXP3− CD4 + T cells post vaccine. In the same subset of infants used for memory phenotyping, utilizing CCR7, CXCR5, CCR4, CCR6 and CXCR3, we were able to parse conventional CD4 + T cells into 6 distinct T-helper (Th) subtypes including T follicular helper cells (Tfh) and 5 different CCR7− Th effector memory subsets (Fig. 2A). Comparatively, RV-specific conventional CD4 + T cells had a significantly higher Th2 bias in their effector memory compartment compared to CMV (p = 0.003, Fig. 2B). CMV-specific responses had the inverse relationship displaying significantly more Th1 polarization than RV (p = 0.03, Fig. 2B). No significant difference was seen in Th0 or Th17 polarization and both RV and CMV specific CD4 + T cells were seldom of the Th17.1 or Tfh subtype (Fig. 2B).

### Response to oral rotavirus vaccination is influenced by pre-existing immunity

**Fig. 3:**
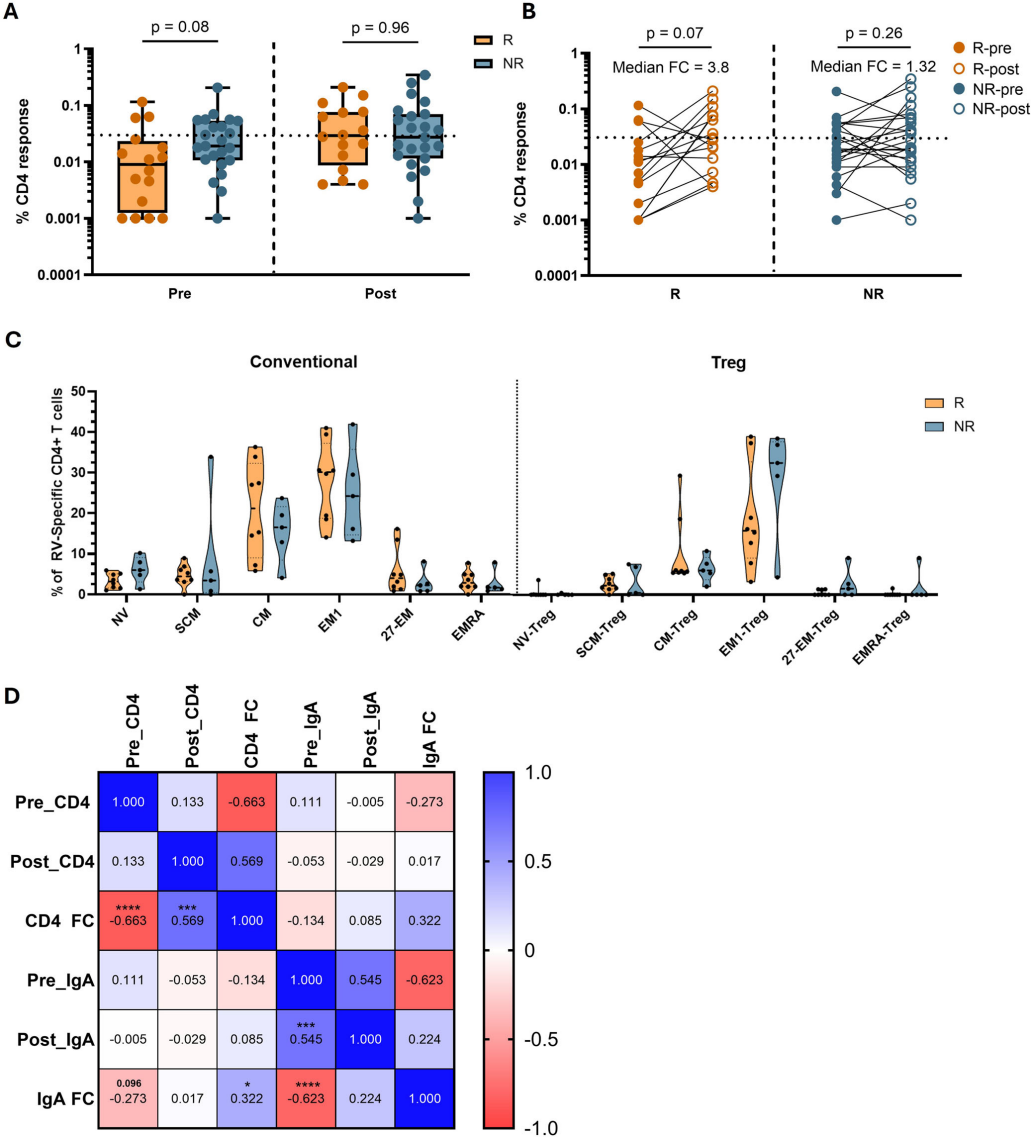
RV-specific CD4 + T cell responses in IgA responders and non-responders. **A** Unpaired analysis of pre-and post-vaccine magnitudes of RV-specific CD4 + T cells identified by Boolean gating compared between IgA responders (n = 16) and non-responders (n = 23). Data are shown with median, quartiles and minimum/maximum values. **B** Paired pre (solid) and post (open) RV-specific CD4 + T cells in IgA responders (n = 16) and non-responders (n = 23). Median fold changes are shown for each group. **C** Unpaired analysis of memory phenotypes of conventional and regulatory CD4 + T cells in T cell responders post vaccine compared between IgA responders (n = 8) and non-responders (n = 5). Data are shown with the median and quartiles. Comparisons between R and NR phenotypes were made by Mann-Whitney tests. **D** Spearman correlation matrix (n = 38) of RV-antigen specific immunity parameters pre- and post-vaccine. Rho values are shown and correlations colored on a gradient from 1 to −1 where values closer to 1 are colored blue (positively correlated) and values closer to -1 colored red (negatively correlated). p values are displayed once per comparison above each respective r value, **** = p < 0.0001. ***p < 0.001, *p < 0.05. Correlations with values approaching significance have their p value displayed numerically. Data points represent individual infants. Horizontal dotted lines represent the limit of sensitivity (LOS) as determined by the median + 2 x standard deviation of a synthetic non-specific myelin oligodendrocyte glycoprotein (MOG) peptide response (A/B = 0.031). RV rotavirus, R RV vaccine serologic responder, NR RV vaccine serologic non-responder, FC fold change, NV naive, SCM stem cell memory, EM effector memory, EMRA effector memory cells re-expressing CD45RA.

Splitting the cohort by IgA response status, pre- to post-vaccination T cell fold change (median = 3.8 for IgA-responders vs 1.3 for non-responders, p = 0.05) and corresponding pre-vaccination CD4 + T cells stimulation indexes (p = 0.04) were significantly different between IgA responder and non-responders, highlighting concordance between the humoral and cellular measures of response (Supplemental Fig. 2C, D). Pre-vaccine CD4 + T cell magnitudes were not significantly different between response groups (p = 0.08, Fig. 3A). IgA seroconversion status had no significant association with the phenotype of regulatory or conventional RV-specific CD4 + T cells post-vaccination in RV vaccine T cell responders (Fig. 3C).

Despite the heterogeneity in post vaccination response types (Table 1), T cell fold change and IgA fold change values were correlated, although the correlation itself was weakly significant (r = 0.32, p = 0.05, Fig. 3D). Pre-vaccine RV-specific CD4 + T cells negatively correlated with CD4 + T cell fold change (r = −0.66, p < 0.0001, Fig. 3D). Similarly, pre-vaccine RV-specific IgA negatively correlated with IgA fold change (r = −0.62, p < 0.001, Fig. 3D). However, despite the correlation between IgA and T cell fold change values, pre-vaccination RV-specific CD4 + T cells did not correlate with IgA fold change (r = −0.27, p = 0.096, Fig. 3D).

### RV-specific CD8 + T cells in the blood are low in magnitude post oral RV vaccine and poorly functional in comparison to CMV-specific cells

**Fig. 4:**
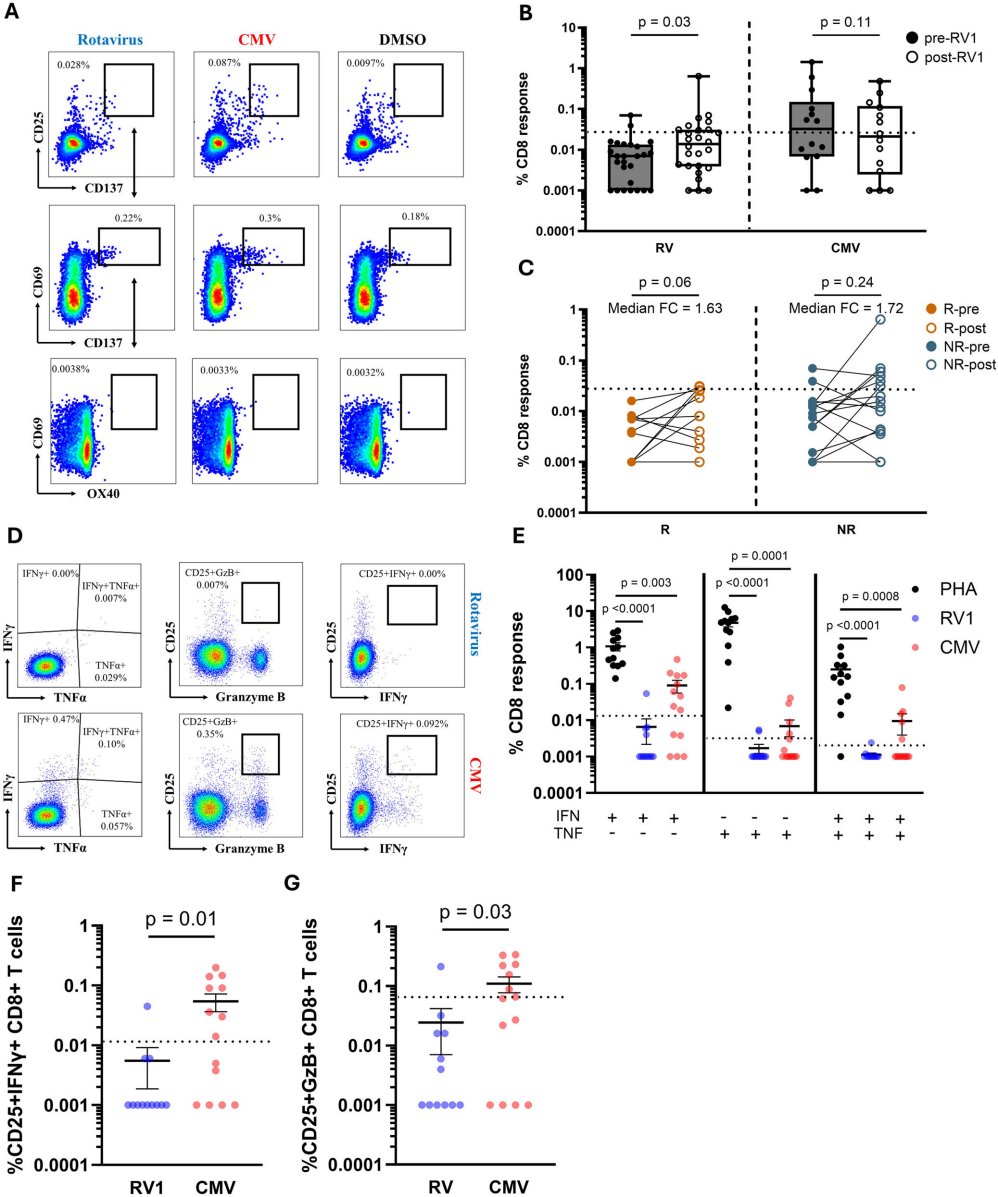
CD8 + T cell responses to RV vaccination in infants compared to CMV. **A** Representative flow cytometry plots showing the gating strategy for determining AIM + CD8 + T cell response using Boolean OR gating. **B** Paired analysis of pre- vs post-vaccine magnitudes of antigen-specific CD8 + T cells identified by Boolean gating. Data are shown with median, quartiles and minimum/maximum values. Magnitudes represent the proportion of RV- (n = 26) or CMV-specific (n = 14) CD8 + T cells - the DMSO vehicle control expressed as a % of total CD8 + T cells (**C**) Matched pairwise comparisons of pre- vs post-vaccine RV-specific CD8 + T cells in IgA responders (n = 11) and non-responders (n = 15). Median FC values are displayed. **D** Representative flow cytometry plots showing the gating strategy for the identification of functional responses in RV- and CMV-specific CD8 + T cells. **E** Unpaired analysis of functional responses of PHA (black), RV (blue) and CMV (red) stimulated CD8 + T cells in T cell responders (n = 11). Data are shown with the mean and standard error of the mean. **F**, **G** Unpaired analysis of CD25+ IFNy+ and CD25+ Granzyme B+ response magnitudes in RV- (n = 12) and CMV-specific (n = 14) CD8 + T cells in T cell responders expressed as a % of total CD8 + T cells. Data are shown with the mean and standard error of the mean. Matched pairwise comparisons in B and C were made by Wilcoxon matched-pairs signed rank tests. Comparisons between IgA responders and non-responders at pre- and post-vaccine and comparison between RV and CMV phenotypes were made by Mann-Whitney. Comparisons across multiple unmatched groups were made by Kruskal-Wallis tests corrected for multiple comparisons by Dunns post hoc test. Data points represent individual infants. Horizontal lines represent the limit of sensitivity (LOS) for each assay as determined by the median + 2 x standard deviation of a synthetic non-specific myelin oligodendrocyte glycoprotein (MOG) peptide response (**B**, **C** = 0.029, **E** IFN + = 0.012, TNFa + = 0.0031, IFN + /TNFa + = 0.0018, **F** = 0.012, **G** = 0.069). PHA phytohemagglutinin-L, RV rotavirus, CMV cytomegalovirus, FC fold change, DMSO dimethyl sulfoxide, R RV vaccine serologic responder, NR RV vaccine serologic non-responder.

We next sought to assess the magnitude and functionality of peripheral blood RV-specific CD8 + T cells in response to RV vaccine. When assayed by AIM, these cells also increased in magnitude significantly after vaccination albeit at very low levels, with the population median not rising above the LOS post vaccination (p = 0.03, Fig. 4A, B). Despite the weak RV-specific response, stronger CMV-specific CD8 + AIM responses were seen in some infants (Fig. 4B). CD8 + T cell responses did not differ between IgA response groups (Fig. 4C). The functionality of RV-specific CD8 + T cells in T cell responders (as determined by CD4+ response status and that passed CD8 QC) was poor in comparison to PHA and CMV-specific cells (Fig. 4D, E). After 24 hours of stimulation, RV-specific CD8 + T cells had significantly lower magnitudes of CD25 + IFNγ + (p = 0.01) and CD25+ Granzyme B+ (p = 0.03) responses compared to CMV-specific cells (Fig. 4F, G). To ensure that the lack of RV response was not a technical phenomenon related to the 24 hour stimulation condition, these findings were further repeated in a complementary analysis (n = 4) after 10 hours of stimulation in which RV-specific cells also demonstrated significantly lower levels of mono- and polyfunctionality compared to CMV-specific cells (p = 0.03, Supplemental Fig. 4). Of the tested participants, 4/4 (100%) had RV-specific monofunctional IFNγ+ responses below the LOS (Supplemental Fig. 4).

### Pre-vaccine serum IL-4 negatively correlates with IgA and T cell fold change responses to RV vaccine

**Fig. 5:**
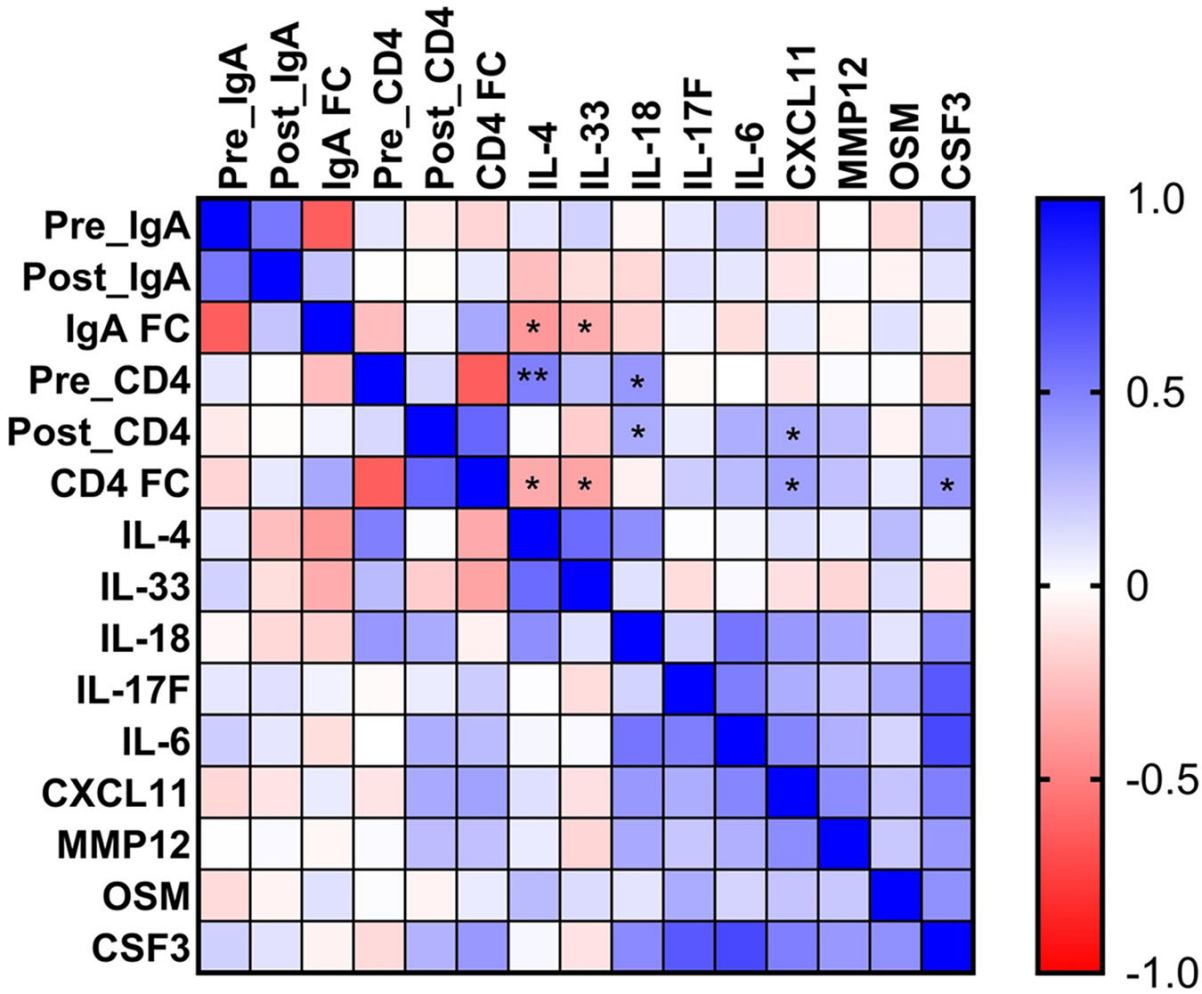
Serum proteome at 2 months of life and pre-RV vaccine. Spearman correlation matrix of RV-antigen specific immunity parameters pre- and post-vaccine and serum proteins identified as significantly up or downregulated in fold change analyses (n = 39). Rho values are shown and correlations colored on a gradient from 1 to −1 where values closer to 1 are colored blue (positively correlated) and values closer to −1 colored red (negatively correlated). p values are displayed only for comparisons between serum proteins and immune parameters and once per comparison, above each respective r value, ** = p < 0.01, * = p < 0.05.

Having profiled the T cell response to RV vaccine and uncovering heterogenous vaccine responses with respect to IgA and T cell response status, we next sought to investigate whether the pre-vaccine serum proteome or bulk peripheral blood T cell compartment correlated with these vaccine response metrics or with country of origin. We used proximity extension assay (OLINK) to quantify 45 serum cytokines, chemokines and growth factors in an exploratory analysis of the 2-month pre-vaccine timepoint. Principal component analysis revealed country level patterns in the serum proteome data, with Peruvian samples grouping independent of Panamanian and US samples (AUC [0.97, 0.90], Supplemental Fig. 5A). This pattern was independent of RV-specific IgA or T cell responses (Supplemental Fig. 5B, C). Next, out of 45 analytes, we identified proteins that were either up or downregulated in IgA or T cell non-responders compared to responders (Supplemental Fig. 6). We identified several significant proteins, though only serum IL-4 was borderline significant after FDR correction (q = 0.078, Supplemental Fig. 6). IL-4 negatively correlated with both RV-specific IgA and T cell fold change, although the correlations themselves were relatively weak (r = −0.40, p = 0.01 and r = −0.33, p = 0.04, respectively, Fig. 5A). However, pre-vaccine serum IL-4 also positively correlated with pre vaccine RV-specific CD4 + T cell magnitude (r = 0.49, p = 0.001).

With proteomic signatures in the 2-month serum correlating with IgA non-response we next investigated the bulk peripheral T cell compartment to determine whether correlations with vaccine response existed within the wider T cell pool. Using a consensus metaclustering approach, we identified 32 unique clusters of T cells in these infants (Supplemental Fig. 7A). A cluster heatmap of relative marker expression across 16 surface phenotyping markers was used to identify statistically relevant clusters (Supplemental Fig. 7B). When comparing the 2-month timepoint to the 6-month timepoint, significant differences were seen in clusters related to increased abundance of terminally differentiated CD4+ and CD8 + T cells as well as CXCR3+ effector memory CD8 + T cells at the 6-month timepoint, signifying an increase in maturity and antigen experience within the T cell compartment consistent with increasing age (Supplemental Fig. 7C, D).

## Discussion

The gold standard immunological measure used in current investigations of RV vaccine efficacy is serum IgA fold change^19,21,33,34^. However, there is evidence of incomplete correlations between peripheral IgA and vaccine effactiveness or stool shedding in the literature^18,20,35^. Notably, there is limited data on RV-specific T cell responses after RV vaccine. Here, we have provided a comprehensive assessment of post vaccination RV-specific T cell immunity. Our results confirm that RV-specific T cell magnitudes in peripheral blood after RV vaccination are low, in line with observations following natural infection^22,26,28,33–35^. However, RV-vaccine does induce significant increases in magnitude in RV-specific CD4 + T cells compared to pre-vaccine levels. Critically, our results provide evidence of heterogeneity in the peripheral response to RV-vaccine, including infants that respond by only IgA or T cells, both or neither. Our findings suggest that IgA alone is an incomplete metric for determination of vaccine immunogenicity. We also show for the first time in the literature long-term T cell maintenance data, demonstrating that RV-specific CD4 + T cell responses in the blood remain present during the period of highest RV infection severity but largely wane by 8 months post RV vaccine.

RV vaccination induced CM, EM, SCM and CD27-EM phenotypes among FOXP3− conventional RV-specific cells, with the bulk of the response being EM. RV-specific FOXP3+ Tregs were mostly of a CM or EM subtype post vaccine suggesting the potential for long term survival. This could have potentially important implications on vaccine effectiveness if regulatory responses in the blood reflect the status of the gut. Effector memory Tregs have the capacity for long term survival and potent immune regulatory propensity upon re-infection^36^. How this may affect RV VE remains unknown. A rapid regulatory response in the gut upon re-infection could blunt the antiviral response to RV and lead to poorer clearance. This hypothesis is supported by observations in other settings such as influenza responses and neo-antigen tumor immunity^37–40^. On the other hand, successful regulatory T cell memory could protect infants from developing symptoms of severe disease, such as diarrhea, by controlling inflammation^36^. In either case, the presence of RV-specific EM Tregs in the blood post vaccine is likely an indicator of a successful immune response to vaccination. Further complimentary work in mice or intestinal tissue models is needed to fully investigate mucosal Treg responses.

The effectiveness of oral RV vaccination is inconsistent across individuals, with variability in response more pronounced in LMICs^6,7,13^. Several theories for this have been suggested, including maternal immune factors such as human milk RV-specific IgA as well as dysbiosis of the infant gut microbiota^33,41,42^. Anti-rotavirus IgA in human milk has been shown to attenuate virus infectivity ex vivo, however there is as yet no solid link between human milk RV-specific IgA titer and VE^33^. Enteric dysbiosis, either due to malnutrition or increased antibiotic use, has been postulated as a key contributor to lower effectiveness of several vaccines^43–46^. However, several studies have found inconsistent correlations with RV-vaccine effectiveness, and Ryan et al. demonstrated no link between antibiotic use and RV vaccine response^34,42,47–49^. One consistent observation is that pre-existing immunity is negatively associated with RV-specific IgA response; our results support this and extend the finding to CD4 + T cell responses^50–53^. We find that pre-vaccination RV-specific CD4+ magnitudes negatively correlated with RV CD4 + T cell fold change in response to vaccination. This could be interpreted in two ways. Firstly, pre-existing immunity may attenuate the live vaccine response. Enteric dysfunction has been associated with increased Tregs that inhibit CD4 + T cell responses locally in the gut^41^. Whether our findings in the peripheral blood reflect the situation in tissue however, is unknown. Second, if an individual already exhibits higher levels of circulating T cells or IgA, responses to vaccination may be mathematically blunted. In either regard, the source of this apparent pre-existing immunity is unknown. The most likely explanation is that these antigen-specific CD4 + T cells represent immunity from infection pre-vaccination, exposure to a sibling or close contact with viral shedding after receiving the live oral RV vaccine. Another possibility is that vaccination that early in life is stimulating highly cross-reactive neonatal immune cells, thus limiting the propensity for lasting T cell memory^54,55^. Our data also shows that higher pre-vaccination RV-specific IgA is negatively correlated with IgA fold change, lending support to the pre-vaccination infection theory. If occurring, pre-vaccination exposure may induce original antigenic sin with respect to the infective strain leading to dampened response to RV1 vaccination. The impact of original antigenic sin on the infant T cell responses to RV vaccine could be further tested by including whole antigen stimulation conditions from other rotavirus genotypes outside of G1P, such as RV5 (RotaTeq—G1, G2, G3, G4). Furthermore, the timing of the first vaccine dose relative to the infection event may result in suboptimal memory responses given that the two exposures are likely occurring close together. The vaccination exposure may therefore be capturing naive T cells still in the process of forming fully mature memory responses. This may in part explain the poor durability and waning of peripheral RV-specific T cell responses. In either case, pre-existing immune response to RV antigen dampens the response to vaccine. High pre-vaccination IgA levels negatively impacting serum IgA seroconversion post-vaccine have been observed by other groups and are particularly prevalent in LMICs^50–53^. Our findings further support this IgA trend and demonstrate that cellular immunity follows the same pattern, a relationship not previously reported.

We found that post-vaccine RV-specific conventional CCR7− CD4 + T cells were biased toward a Th2 chemokine receptor profile. Though this result is suggestive in the absence of matching antigen specific mechanistic data, the pattern was recapitulated within the broader serum proteome. In an exploratory analysis of infant serum, we found a type 2 signature of increased IL-4 at the timepoint immediately preceding vaccination in vaccine non-responders. Lower Th1:Th2 ratios and poor type-1 functionality is well described in neonates and has been observed in other human and murine infant vaccination contexts^56–58^. A recent study found evidence of Th2 CD4+ memory T cell induction in the acute phase of RV+ diarrhea but not RV- diarrhea within the bulk T cell pool^23^. Although most of the infants in the referenced study had been previously vaccinated with Rotarix™ (RV1), our data represents the first time this same pattern of Th2 memory has been observed in RV1-vaccine strain specific T cells post vaccine^23^. Given that most of the infections reported by Banda et al. occurred at 12 months of life, corresponding to our T cell waning data, the notable drop in vaccine-derived immunity in our cohort becomes more important^23^. Indeed, if there are individual differences with respect to T cell waning, perhaps this could point to an increased chance of infection in those individuals. The same study also provided more evidence of poor functionality among RV VP6-specific T cells^23^. Our data also shows poor functionality among RV1 vaccine strain-specific T cells. This bias toward a type-2 phenotype may explain the poor type-1 antiviral functionality of peripheral blood RV-specific T cells. The phenomenon of Th2 response skewing has also been demonstrated in infants <3 months of age infected with respiratory syncytial virus (RSV)^59^. Given that the RV vaccine was administered at 2 and 4 months of age in our cohort, the data may be pointing to temporal biases with respect to T cell response, with earlier age of exposure inducing poorly Th1 functional and low memory propensity responses. However, given that strong type 1 antiviral responses to CMV can be seen as early as 2 months of life, this pattern is also likely antigen dependent. CMV is a more persistent and accessible antigen as compared to RV, which is likely more restricted to the intestinal mucosa. This may explain the difference in response magnitude. However, this comparison provides important context for the low RV response, confirming that peripheral antigen specific responses to RV in infants are low, either reflecting a phenomenon inherent to that particular antigen in infants or a more tissue resident response. Further study of infant T cell development across early life and its impact on multiple bacterial, viral, fungal and parasitic antigen responses is needed.

We show that RV-specific CD8 + T cell responses in peripheral blood are low in magnitude, barely detectable and poorly functional, in line with findings from studies of responses following natural infection^22,26,28,31^. However, compared to RV-specific cells, CMV-specific CD8 + T cells in these infants were more functionally capable, producing detectable amounts of IFNγ, Granzyme B and exhibiting polyfunctionality. The CD8+ functionality of CMV specific cells seen in our cohort is in agreement with previous reports demonstrating that infants mount effective CD8 (but not CD4) responses to CMV^60–65^. Furthermore, these findings agree with previous work in the natural infection setting, where CMV was used to compare functional responses to RV infection^22^. However, this may not reflect the tissue resident memory response. Results from immunodeficient mouse models clearly demonstrate non-redundancy with respect to the CD8+ response to RV infection^24,25^.

We have shown evidence of CD4 + T cell induction by RV vaccine in the peripheral blood but that this peripheral memory response wanes by 8 months post. Crucially, the waning of peripheral CD4 + T cell memory is specific to individuals, with some displaying complete loss of detectable response whilst others maintain responses above the limit of sensitivity. This could reflect a more tissue centric memory response or poor long-term memory induction stemming from the stimulation of early life T cells by RV vaccine. Next steps for further investigation include extended follow-up periods, use of tissue-specific markers to identify and track resident memory populations, and comparative TCR sequencing and fate-mapping in both humans and murine models.

This study has several notable limitations including limited sample size (n = 39), due primarily to sample availability. Many comparisons involve small sample sizes making statistical comparisons underpowered. Low antigen-specific event counts limit the stability of the detailed phenotypic analysis, although care was taken to remove samples with <10 events. How well peripheral T cell memory to RV reflects that of intestinal tissue resident cells is unknown. Working in human infants results in limited access to tissue samples. Complementary experiments in infant mouse vaccination models or intestinal organoids would provide more data. Sample availability to generate sufficient PBMCs to comprehensively study T cell responses in infants remain a limitation for vaccine studies. Infants with gastrointestinal symptoms that occurred during the study period did not have stool tested for RV, however, all participants who reported symptoms were negative for RV-specific IgA at 2 months of age prior to vaccination. Participants were also not tested for CMV excretion in saliva or urine, nor were they evaluated serologically. Only RV1-specific peptides were evaluated for T cell functionality and memory recall. Though this was by design, given our cohorts’ exclusive vaccination with RV1, it limits the ability to assay response breadth across multiple epitopes. Given our observation of pre-vaccine T cell responses, future comparative stimulation with RV5 or wild-type virus could elucidate source and cross-reactivity of the pre-existing immunity. The use of peptide megapools limits our ability to assess nonlinear conformational epitopes which could be addressed by supplementary stimulation with whole antigen. Waning of peripheral CD4 + T cell responses is suggested in our cohort, however the low n (n = 8) of this assay, due to sample availability, makes any conclusions unreliable despite the statistical significance. This assay should be repeated in a larger cohort. In the absence of intracellular IL-4 or IL-5 data, the skewing of CD4 + T cells toward Th2 based on chemokine receptor profiles is suggestive rather than mechanistic. Given the difficulty in assaying type 2 cytokines in infants, complimentary experiments, such as transcription factor staining, could further bolster these findings.

In conclusion, we demonstrate that RV vaccine elicits heterogenous peripheral immune responses in infants from three different countries of varying socioeconomic status. RV vaccine elicits significant RV-specific CD4 + T cell responses in the blood. Despite strong memory phenotype induction, including stem-cell memory, central memory and effector memory subtypes, these responses wane 8 months after vaccination to pre-vaccine levels. The rapid waning of peripheral T cell immunity suggests the response to vaccine is largely tissue centric and that peripheral memory generation is suboptimal. Whether differences in waning and RV-specific T cell durability are linked to future infection susceptibility remains unknown, as does the RV-vaccine specific TCR repertoire and epitope immunodominance in this age group. Both would be interesting avenues of future research to determine the long-term impact of RV-vaccine T cell immunity in the absence of tissue data. We provide further evidence that infants with pre-existing immunity do not demonstrate significant magnitude increase above their respective pre-vaccine levels, showing this pattern both in peripheral RV-specific IgA and, for the first time, in peripheral RV-specific CD4 + T cells. However, quantitatively, responses are similar to post-vaccine values of those without pre-existing immunity, perhaps suggesting an immunological plateau with respect to peripheral responses that is reached by either infection or vaccination. In either case, peripheral T cell responses and their waning remain important considerations for future vaccine strategies.

## Methods

### Study design

Infants were enrolled prior to their first RV vaccine dose at Kaiser Permanente (Los Angeles, USA), Centro Materno Infantil Tahuantinsuyo Bajo, Independencia, Universidad Peruana Cayetano Heredia (UPCH; Lima, Peru), and Hospital del Niño Doctor Jose Renan Esquivel (Panama City, Panama), using a convenience recruitment strategy. Infants enrolled in the study were vaccinated with the oral live, attenuated monovalent rotavirus vaccine (RV1, Rotarix™, GlaxoSmithKline) at 2 months and 4 months according to the immunization schedule of each country. Blood samples were obtained at 2 (pre-vaccination), 6 (post-vaccination), and 12 months of life (Supplemental Fig. 8).

Demographic characteristics were collected by survey at enrollment, and medical history questions were obtained at each visit. Written informed consent was obtained from the parents/legal guardians of all participants. CHLA was the primary coordinating site, and ethics approval was obtained by the CHLA institutional review board (IRB#: CHLA-16-00229). In addition, approval was obtained by ethics committees at each participating site. Ethical guidelines were strictly followed at all sites.

### Sample processing, storage and shipment

Serum was isolated from serum separator tubes by centrifugation, aliquoted, and stored at −80 °C for shipment. PBMCs were isolated from EDTA tubes using Ficoll-Paque density gradient centrifugation. Briefly, blood samples were mixed 1:1 with phosphate-buffered saline (PBS) solution (Gibco, ThermoFisher Scientific) and transferred to Leucosep tubes (Greiner Bio-One). After centrifugation, buffy coat layers were transferred to new 50 mL tubes and washed twice with PBS. PBMCs were counted and resuspended in 10% dimethyl sulfoxide (DMSO) (ThermoFisher) in fetal bovine serum (FBS) (Genesee Scientific) for storage. Aliquots were immediately placed in a Mr. Frosty isopropyl alcohol cell cooler and moved to −80 °C for step-down cooling at −1 °C/minute. After 24 hours, cells were moved to liquid nitrogen tanks for long-term storage. Panamanian and Peruvian serum, plasma, and PBMC samples were periodically shipped to UCSD on dry ice and liquid nitrogen dry shippers, respectively, for analysis. To ensure sample integrity, the internal temperature of the shipping container was continually monitored throughout the transfer process.

### Serum anti-rotavirus IgA

RV-specific serum IgA was measured using an enzyme-linked immunosorbent assay (ELISA). Briefly, high binding 96-well plates (Corning) were coated in rabbit rotavirus-specific hyperimmune serum (kindly provided by Dr. Baoming Jiang, Centers for Disease Control and Prevention) at a concentration of 1:10,000 in coating buffer (pH 9.6) and incubated overnight at 4 °C. After 4 washes with PBS-0.05% Tween20 (PBS-T), plates were incubated with 50 µL Rotarix™ vaccine (GSK Biologicals, stock titer 6.67 × 10^5^ CCID50–50% Cell Culture Infective Dose) diluted 1:5000 in diluent buffer (1% skim milk in PBS-T) for 1 hour at 37 °C and shaking at 270 rotations per minute (final concentration of 133 CCID50/mL). The plates were then washed and incubated with 200 µL blocking solution (3% skim milk in PBS-T) for 1 hour. After blocking, plates were incubated with 1:20 diluted serum samples in diluent buffer and a 1:10 diluted positive standard control serially diluted 1:3. Plates were then washed and stained with goat anti-human IgA secondary antibody (Southern Biotech, Birmingham, USA) diluted 1:5000, and reactions developed with 3,3’,5,5’-tetramethylbenzidine (TMB). The plate reaction was quenched with 2 M sulfuric acid, and optical density (OD) values were measured at 450 nm immediately after. A positive standard control consisting of pooled serum derived from 5 high RV-IgA adult donors from a rotavirus endemic country was used for assay standardization. The positive pool was assigned an arbitrary value of 100 AU/mL and 3-fold-serially diluted to generate a standard curve for each plate. The AU/mL for each test sample was calculated by interpolating the OD value against this standard curve. Seroconversion was defined as a ≥ 3-fold change in post-vaccination infant serum IgA compared with pre-vaccination serum IgA. A seropositivity threshold of ≥20 AU/mL for serum IgA was established based on the 1.5 × IQR plus Q3 of serum IgA levels in pre-vaccination samples.

### Peptide selection and production

To study T cell responses against CMV and RV, two peptide pools (Megapools; MP) were prepared following the MP approach, previously outlined as a comprehensive method for analyzing T cell responses across diverse epitopes and populations^66^. The CMV pool encompassed a total of 187 peptides and was based on previously reported experimental class II epitopes^67^. The RV pool encompassed a total of 225 peptides designed after the G1P[8] RV strain found in the RV1 vaccine which contains 12 proteins including 6 structural and 6 non-structural proteins. Proteins were scanned for the presence of predicted HLA class II promiscuous binding peptides using the combined HLA binding and immunogenicity predictor tool, utilizing the Immune Epitope Database and Analysis Resource (IEDB). Briefly, the prediction of peptides was established by the 7-allele HLA class II restricted method (PMID: 25862607). This methodology is a widely used computational approach for predicting promiscuous T cell epitopes in human populations, especially when an individual’s specific HLA typing is unavailable. Specifically, it predicts T cell epitopes by focusing on the median binding strength of a representative set of common HLA Class II alleles: DRB1*03:01, *07:01, *15:01, DRB3*01:01, *02:02, DRB4*01:01, and DRB5*01:01. It efficiently identifies peptides likely to bind to many HLA types, helping predict population-level immune responses by selecting peptides with a low median consensus percentile rank (e.g., ≤ 20.0) as promising binders and potential epitopes. In the case of the RV peptide pool design, we selected a more stringent combined median percentile rank with a cut-off of 10.0 for each individual RV protein using 15-mer peptides overlapping by 10 residues. Additional filtering using an epitope cluster analysis was performed to include unique peptides across all proteins and to encompass experimentally validated HLA class II restricted peptides retrieved from IEDB search engine (Supplementary Data 1). All peptides were synthesized by TC peptide lab (San Diego, CA) on a small (1 mg) crude scale (>85% purity). Peptides were pooled, lyophilized and resuspended at a final concentration of 2 mg/mL in DMSO.

### Combined activation induced marker (AIM) and intracellular cytokine staining (ICS) assay

Cryopreserved PBMCs were rapidly thawed and rested for 4 hours in RH media (RPMI + 5% Human Serum + 1% pen/strep containing 1/5000 benzonase (Millipore 71206-25KUN)) at 37 °C. After resting, cells were pelleted, counted and aliquoted into wells of a 96-well plate at a density of 1 × 10^6^ cells per well in 100 µL of RH. Conditions tested included, as a minimum, a phytohemagglutinin (PHA) positive control, Dimethyl sulfoxide (DMSO) negative control and rotavirus MP. A CMV MP condition was also added in samples with sufficient cell quantity. Cells were pre-stained in culture with the pre-stain antibody mix (Supplemental Table 1) and incubated at 37 °C for 20 minutes. Without washing, cells were stimulated with respective mitogens or condition specific peptide MPs in a final volume of 200 µL for 20 hours, incubated at 37 °C (PHA was added at a final concentration of 10 µg/mL, DMSO and antigen specific peptide MPs were added at a final concentration of 1 µg/mL). At hour 20, AIM markers (Supplemental Table 1) were added alongside protein transport inhibitors (BD GolgiPlug™ containing brefeldin A and BD GolgiStop™ containing monensin, cat #555029 and 554724, respectively) and the cells incubated for a further 4 hours. After 24 hours of stimulation, cells were washed twice with PBS and stained with Zombie UV fixable viability dye in a final volume of 50 µL (Biolegend, 423108) for 15 minutes. Subsequently and without washing, 50 µL of 10% TruStain FcX blocking solution was added for a further 15 minutes in a final volume of 100 µL. After incubation, cells were washed with PBS and resuspended in 100 µL of surface stain cocktail (Supplemental Table 1). 50 µL of brilliant stain buffer (Becton Dickinson, 566349) was added to each sample for a total final volume of 150 µL. Samples were stained for 30 minutes at room temperature. After surface staining, cells were washed with PBS and fixed by resuspending in 200 µL of FOXP3 fixation/permeabilization buffer (Thermo Fisher Scientific, 00-5523-00) as per the manufacturer’s instruction. After 30 minutes of incubation at room temperature, cells were washed in 200 µL of 1x permeabilization buffer (Invitrogen, 00-5523-00) and resuspended in 50 µL of ICS blocking solution (permeabilization buffer + 10% human serum) and incubated for 15 minutes at room temperature. After incubation and without washing, 50 µL of intracellular cocktail (Supplemental Table 1) was added to each sample for a final volume of 100 µL and incubated for a further 30 minutes with 10% brilliant stain buffer added. After intracellular staining, cells were washed twice with FACS buffer (PBS with 2% FBS and 0.01% NaN3). Cells were resuspended in an appropriate volume of FACS buffer and ran immediately on the CyTEK AURORA 5 L. Data was unmixed using a combination of bead and cell reference controls, as outlined in Supplemental Table 2, and post-unmixing compensation (Supplementary Table 3) applied where required. Alternative to the 24-hour stimulation protocol (20 hours of normal stimulation + 4 hours of incubation with AIM markers and protein transport inhibitors), stimulation was carried out for a total of 10 hours with 6 hours of normal stimulation plus 4 hours of incubation with AIM markers and protein transport inhibitors.

### Flow cytometry data analysis

Unmixed and compensated data files were exported into FlowJo v10.10.0 for gating analysis. Single-stain cell-based controls were used to generate the compensation matrix (Supplemental table 3). RV and/or CMV samples were omitted from analysis if they had fewer than 80,000 CD4s and/or 40,000 CD8s and/or had a significant number of dead cells (>25% of total lymphocytes). Samples were pre-gated on single, live, CD3+ lymphocytes and separated into CD4+ and CD8+ fractions (Supplemental Fig. 9). AIM + CD4 and CD8 T cell responses were determined by Boolean OR gating across a spectrum of AIM marker combinations. Upper boundaries of negative populations for each marker of interest were determined by FMO controls within PHA stimulated PBMCs and used as a guide for final gating positions (Supplemental Figs. 10–14). Functional gates were also confirmed in the PHA control first. Memory phenotyping gates were positioned first for the bulk CD4 (or CD8) T cells and then the AIM+ cells overlayed. AIM+ and functional responses were determined both by stimulation index (Ag+ magnitude/DMSO magnitude) and by DMSO subtraction. Limits of sensitivity for the DMSO-subtracted magnitude readout were determined in a subset of PBMCs from five 2-month-old infants as the median + 2 x standard deviation of a MOG synthetic non-specific peptide control (JPT, Swiss-Prot ID: Q16653, aa 30-154). A minimum threshold for stimulation index was set at 2 and confirmed by the MOG control. T cell responder status was determined as a FC ≥ 2 when comparing the post-vaccine DMSO-subtracted CD4 + T cell magnitude to the pre. T cell responders were only confirmed as such if the post-vaccine DMSO-subtracted magnitudes were >LOS and with stimulation indexes ≥2. CD4+ phenotyping in Figs. 1D–G and 2A, B was only performed in samples with >10 antigen-specific events to not artificially inflate magnitude proportion data.

### Batch normalization and high dimensional analysis of flow cytometry data

FlowSOM metaclustering and dimensionality reduction analysis was achieved within an OMIQ workflow. Briefly, compensated and cleaned FCS files were imported and markers arcsinh transformed (cofactor = 150). The dataset was subsampled to include 5000 events from each sample to limit computational burden. Data was batch normalized using CytoNorm utilizing consistently run batch controls as the training set with data normalized to FlowSOM consensus metaclusters k = 30 with Euclidean distance. Sample data was then re-clustered using the normalized marker values using FlowSOM consensus metaclustering k = 30 with Euclidean distance. Normalized metaclusters were analyzed manually and clusters split, merged or deleted to generate the final cluster set for analysis. The data was visualized using opt-SNE (max iterations = 1000, opt-SNE end = 5000, perplexity = 30, theta = 0.5, components = 2, verbosity = 25). Clusters were defined only if >100 events were present. Statistical analysis between metadata was performed using edgeR. Cluster identification was based on cluster heatmaps of relevant normalized markers using Euclidean distance as the metric for clustering.

### Serum proteomics by proximity extension assay (OLINK^TM^)

Proximity extension assay (Target-48 panel) was used to quantify serum cytokines, chemokines and growth factors. Serum samples were sent to LuminoDx (San Diego, CA), an OLINK (Thermo Fisher Scientific, Waltham, MA) commercial partner. The Target-48 Cytokine Panel quantified 45 individual proteins using 2 µL of serum sample per test. Internal controls included a duplicate negative (calculation of limit of detection (LOD)), triplicate calibrator (normalization) and triplicate sample control (intra- and inter-plate variation). Normalized Protein eXpression (NPX) values were used for downstream analysis after QC. Readouts consistently above or below the limit of quantification were omitted from the analyses. Proteomic signatures of rotavirus vaccine IgA non-responders were compared to responders by fold change analysis. The significant up- or down-regulation of serum proteins was determined by a log2FC threshold and non-parametric p values < 0.05 (Mann-Whitney) and FDR correction.

### Statistical analysis

Given the lack of similar studies in the literature, power analysis was based on preliminary data which was used to calculate an effect size of dz = 0.4455329 for the pre- vs post-vaccine RV-specific CD4 T cell magnitude change. A power analysis was calculated for a Wilcoxon signed-rank test, one tailed with α error probability set at 0.05 and power set at 0.95 for the calculated effect size of 0.4455329. The power analysis calculated 59 samples as the optimal n.

For categorical variables; comparisons between sex were made by a two-sided Fishers exact test. Comparisons between country, race, and ethnicity were made using a Chi squared test. Continuous variables are displayed with median, interquartile range (IQR) and minimum/maximum values. Paired comparisons were performed using the Wilcoxon matched pairs signed rank test. Paired comparisons across >2 groups were performed by the Friedmans test with Dunns post hoc test for multiple comparisons. Unpaired comparisons across two groups were performed using the Mann-Whitney test. Unpaired comparisons across >2 groups were performed using the Kruskal-Wallis test with Dunns post hoc test for multiple comparisons. Two-tailed P values are displayed. Correlation analysis was performed using two-tailed Spearman’s test with the confidence interval set at 95%. Variables used in principal component analyses were center scaled prior to ordination. Country level differences identified by the PCA were analyzed by multiple logistic regression with country as the dependent variable and PC scores for PC1 and PC2 across the 39 individuals used as independent variables. Statistical analyses were performed using GraphPad Prism version 10.4.2 (San Diego, CA) and RStudio version 2024.4.2.764 (Boston, MA).

## Supplementary information

Supplementary information

Supplementary informationdata

Supplementary informationdata

## Data Availability

Source data for all figures, including those in the supplement, are provided with this paper. Full data for rotavirus and CMV responses are available at the Open Science Framework as is the code and data used to generate fold change volcano plots 10.17605/OSF.IO/D8SJ2. FCS files used in the analyses have been deposited in the following Zenodo databases, 10.5281/zenodo.17363217, 10.5281/zenodo.17364328, 10.5281/zenodo.17364765.
