# Supplementary material for: Peripheral rotavirus-specific T-cell responses following monovalent oral rotavirus vaccine in infants
Source: NPJ Vaccines. 2026 Mar 5;11:83. doi: 10.1038/s41541-026-01405-z (PMC13083868; doi:10.1038/s41541-026-01405-z)
Supplement: Supplementary file 1 — Supplementary information [file 41541_2026_1405_MOESM1_ESM.pdf]

Supplementary Information

ANTIBODY	IDENTIFIER
**Antibody cocktails**	
Pre-stain antibody mix	
Anti-human CD40L pure functional grade	Cat# 130-094-133
Anti-human CCR6-FITC (G034E3)	Cat# 353412
Anti-human CXCR5-Alexa Fluor 700 (J252D4)	Cat# 356916
Anti-human CCR7-APC (G043H7)	Cat# 353214
Anti-human CXCR3-BV421 (1C6/CXCR3)	Cat# 562558
Anti-human CCR4-BUV615 (1G1)	Cat# 613000
AIM antibody mix	
Anti-human CD69-APC-Cy7 (FN50)	Cat# 310914
Anti-human CD154-BV711 (24-31)	Cat# 752850
Anti-human PD-1-Super Bright 436 (J43)	Cat# 62-9985-82
Anti-human CD25-Real Blue 705 (2A3)	Cat# 570250
Anti-human OX40-PE (Ber-ACT35)	Cat# 350004
Anti-human CD137-BV650 (4B4-1)	Cat# 309828
Live/Dead	
Zombie UV Fixable viability kit	Cat# 423108
Surface stain antibody mix	
Anti-human CD95-PE-Cy5 (DX2)	Cat# 559773
Anti-human CD3-APC-Fire 810 (SK7)	Cat# 344858
Anti-human CD38-PerCP-eFluor 710 (HB7)	Cat# 46-0388-42
Anti-human CD57-BV510 (QA17A04)	Cat# 393314
Anti-human HLADR-BV570 (L243)	Cat# 307638
Anti-human CD28-Real Blue 780 (L293)	Cat# 755600
Anti-human CD4-BV750 (SK3)	Cat# 566356
Anti-human CD45RO-BUV563 (UCHL1)	Cat# 748369
Anti-human CD27-BUV661 (M-T271)	Cat# 741609
Anti-human CD8-BUV805 (SK1)	Cat# 612889
ICS antibody mix	
Anti-human Granzyme B-Real Blue 545 (GB11)	Cat# 569291
Anti-human IFNγ-Real Yellow 775 (B27)	Cat# 571387
Anti-human FOXP3-PE-Dazzle 594 (206D)	Cat# 320126
Anti-human IL-17A-Pacific Blue (BL168)	Cat# 512312
Anti-human TNFα-BV785 (MAb12)	Cat# 502948
Anti-human IL-2-BUV737 (MQ1-17H12)	Cat# 612836

Supplemental Table 1: Antibody master mixes

Antigen	Fluorophore	Clone	Vendor	Cat#	Titration/1 x10^6 cells/100 µL	Optimal Reference Control	Events for Reference Control
CCR6	FITC	G034E3	Biolegend	353412	1/500	Ultracomp Beads	30,000
Granzyme B	RB545	GB11	Beckton Dickinson	569291	1/500	Cells-PHA	100,000
CD25	RB705	2A3	Beckton Dickinson	570250	1/100	Cells-PHA	100,000
CD38	PerCP-eFluor 710	HB7	Invitrogen	46-0388-42	1/50	Cells-PHA	80,000
CD28	RB780	L293	Beckton Dickinson	755600	1/100	Cells-PHA	80,000
OX40	PE	ACT35	Biolegend	350004	1/200	Cells-PHA	100,000
FOXP3	PE-Dazzle 594	206D	Biolegend	320196	1/100	Ultracomp Beads	30,000
CD95	PE-Cy5	DX2	Becton Dickinson	559773	1/50	Cells-PHA	50,000
IFNγ	RY775	B27	Beckton Dickinson	571387	1/100	Cells-PMA	80,000
CCR7	APC	G043H7	Biolegend	353214	1/100	Cells-PHA	100,000
CXCR5	Alexa Fluor 700	J252D4	Beckton Dickinson	356916	1/100	Cells-PHA	100-200,000
CD69	APC-CY7	FN50	Biolegend	310914	1/200	Cells-PHA	80,000
CD3	APC-Fire 810	SK7	Biolegend	344858	1/100	Cells-PHA	50,000
CXCR3	BV421	1C6	Beckton Dickinson	562558	1/100	Ultracomp Beads	30,000
PD-1	Super Bright 436	J43	Invitrogen	62-9985-82	1/200	Cells-PHA	100,000
IL-17A	Pacific Blue	BL168	Biolegend	512312	1/200	Ultracomp Beads	30,000
CD57	BV510	QA17A04	Biolegend	393314	1/50	Cells-PHA	80,000
HLADR	BV570	L243	Biolegend	307638	1/100	Cells-PHA	80,000
CD137	BV650	4B4-1	Biolegend	309828	1/100	Cells-PHA	100,000
CD154	BV711	24-31	Beckton Dickinson	752850	1/500	Cells-PHA	100,000
CD4	BV750	SK3	Beckton Dickinson	566355	1/500	Cells-PHA	50,000
TNFα	BV785	mAb11	Biolegend	502948	1/100	Cells-PMA	80,000
LD	Zombie UV		Biolegend	423107	1/2000	Cells-PHA Deadspike	100-200,000
CD45RO	BUV563	UCHL1	Beckton Dickinson	748369	1/200	Cells-PHA	80,000
CCR4	BUV615	1G1	Beckton Dickinson	613000	1/500	Ultracomp Beads	30,000
CD27	BUV661	m-t271	Beckton Dickinson	741609	1/100	Cells-PHA	80,000
IL-2	BUV737	MQ1-17H12	Beckton Dickinson	612836	1/100	Cells-PMA	80,000
CD8	BUV805	SK1	Beckton Dickinson	612889	1/200	Cells-PHA	50,000

Supplemental Table 2: Antibody dilutions and reference control

Features	Zombie UV-A	BUV563-A	BUV615-A	BUV661-A	BUV737-A	BUV805-A	BV421-A	Super Bright 436-A	Pacific Blue-A	BV510-A	BV570-A	BV650-A	BV711-A	BV750-A	BV785-A	FITC-A	RB545-A	PerCP-eFluor 710-A	RB705-A	RB780-A	PE-A	PE-Dazzle 594-A	PE-Cy5-A	RY775-A	APC-A	Alexa Fluor 700-A	APC-Cy7-A	APC-Fire 810-A	AF-A
Zombie UV-A	100	0	0	0	0	0	0	0	0	0	0	0	0	0	0	0	0	0	0	0	0	0	0	0	0	0	0	0	0
BUV563-A	0.49	100	0	0	0	0	0	0	0	0	0	0	0	0	0	0.65	1.08	0	0	0	8.5	0	0	0	0	0	0	0	0
BUV615-A	0	0	100	0	0	0	0	0	0	0	0	0	0	0	0	0	0	0	0	0	0	0	0	0	0	0	0	0	0
BUV661-A	0	0	0	100	0	0	0	0	0	0	0	0	0	0	0	0	0	0	0	0	0	-2.39	0	0	0	0	0	0	0
BUV737-A	0	0	0	0	100	0	0	0	0	0	0	0	0	0	0	0	0	0	0	0	0	0	0	0	0	0	0	0	0
BUV805-A	0	0	0	0	0	100	0	0	0	-0.56	-0.56	0	0	0	0	-0.74	-0.72	0	0	0	-0.59	-0.93	0	0	0	0	0	0.46	0
BV421-A	0	0	0	0	0	0	100	0	0	0	0	0	0	0	0	0	0	0	0	0	0	0	0	0	0	0	0	0	0
Super Bright 436-A	0	0	0	0	0	0	0	100	0	0	0	0	0	0	0	0	0	0	0	0	0	0	0	0	0	0	0	0	0
Pacific Blue-A	0	0	0	0	0	0	0	0	100	0	0	0	0	0	0	0	0	0	0	0	0	0	0	0	0	0	0	0	0
BV510-A	0.44	1.48	0	0.11	0	0	0	0	0.61	100	0.78	0.56	0	0	0	0	0.38	0	0	0	0.49	0	0	0	0	0	0	0	0
BV570-A	0	0	0	0	0	0	0	0	0	0	100	0	0	0	0	0	0	0	0	0	1.69	0	0	0	0	0	0	0	0
BV650-A	0	0	0	0	0	0	0	0	0	0	0	100	0	0	0	0	0	0	0	0	0	0	0	0	0	0	0	0	0
BV711-A	0	0	0	0	0	0	0	0	0	0	0	0	100	0	0	0	0	0	0	0	0	0	0	0	0	0	0	0	0
BV750-A	0	0	0	0	0	0	0	0	0	0	0	0	0	100	0	0	0	0	0	0	0	-1.3	0	0	0	0	0	0	0
BV785-A	0	0	0	0	0	0	0	0	0	0	0	0	0	0	100	0	0	0	0	0	0	0	0	0	0	0	0	0	0
FITC-A	0	0	0	0	0	0	0	0	0	0	0	0	0	0	0	100	0	0	0	0	0	0	0	0	0	0	0	0	0
RB545-A	0	0	0	0	0	0	0	0	0	0	0	0	0	0	0	0	100	0	0	0	0	0	0	0	0	0	0	0	0
PerCP-eFluor 710-A	0	0	0	0	0	0	0	0	0	0	0	0	0	0	0	0	0	100	0	0	0	0	0	0	0	0	0	0	0
RB705-A	0	0	0	0	0	0	0	0	0	0	0	0	0	0	0	0	0	0	100	0	0	0	0	0	0	0	0	0	0
RB780-A	0	0	0	0	0	0	0	0	0	0	-0.86	0	0	0	0	-2.07	-1.24	0	0	100	0	-1.97	0	0	0	0	0	0	0
PE-A	0	0	0	0	0	0	0	0	0	0	0	0	0	0	0	0	0	0	0	0	100	0	0	0	0	0	0	0	0
PE-Dazzle 594-A	0	0	0	0	0	0	0	0	0	0	0	0	0	0	0	0	0	0	0	0	0	100	0	0	0	0	0	0	0
PE-Cy5-A	0	0	0	0	0	0	0	0	0	0	0	0	0	0	0	0	0	0	1.72	0	0	0	100	0	0	0	0	0	0
RY775-A	0	0	0	0	0	0	0	0	0	0	0	0	0	0	0	0	0	0	0	0	0	0	0	100	0	0	0	0	0
APC-A	0	0	0	0	0	0	0	0	0	0	-0.68	0	0	0	0	-1.14	0	0	0	0	0	-1.22	0	0	100	0	0	0	0
Alexa Fluor 700-A	0	0	0	0	0	0	0	0	0	0	0	0	0	0	0	0	0	0	0	0	0	0	0	0	0	100	0	0	0
APC-Cy7-A	0	0	0	0	0	0	0	0	0	0	0	0	0	0	0	0	0	0	0	0	0	0	0	0	0	0	100	0	0
APC-Fire 810-A	0	0	0	0	0	0	0	0	0	0	0	0	0	0	0	0	0	0	0	0	0	0	0	0	0	0	0	100	0
AF-A	0	0	0	0	0	0	0	0	0	0	0	0	0	0	0	0	0	0	0	0	0	0	0	0	0	0	0	0	100

Supplemental Table 3: Compensation matrix applied to flow cytometry samples.

Supplementary Data 1: RV peptide pool design.

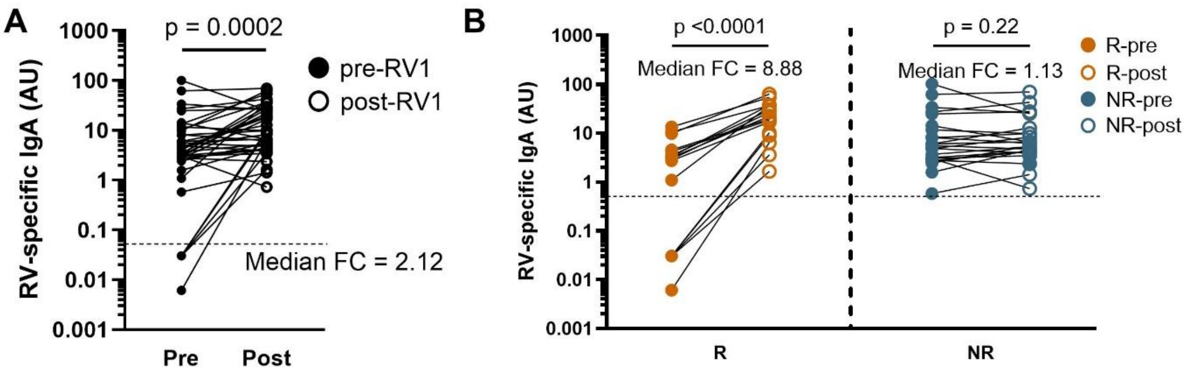

Supplemental Figure 1: Serum IgA responses pre- vs post-RV vaccine series. **A.** Pairwise analysis of serum RV-specific IgA responses in the full cohort measured by ELISA with values displayed in arbitrary units (AU). **B.** Paired comparisons of pre- vs post-vaccine RV-specific serum IgA in IgA responders (n = 16) and non-responders (n = 23). Pre- and post-vaccine were compared using Wilcoxon matched-pairs signed rank tests. The horizontal dotted line represents the limit of detection (0.05 AU/mL) calculated as the mean of the blank wells + 3 standard deviations. Data points represent individual infants. Abbreviations: RV, rotavirus; R, RV vaccine serologic responder; NR, RV vaccine serologic non-responder.

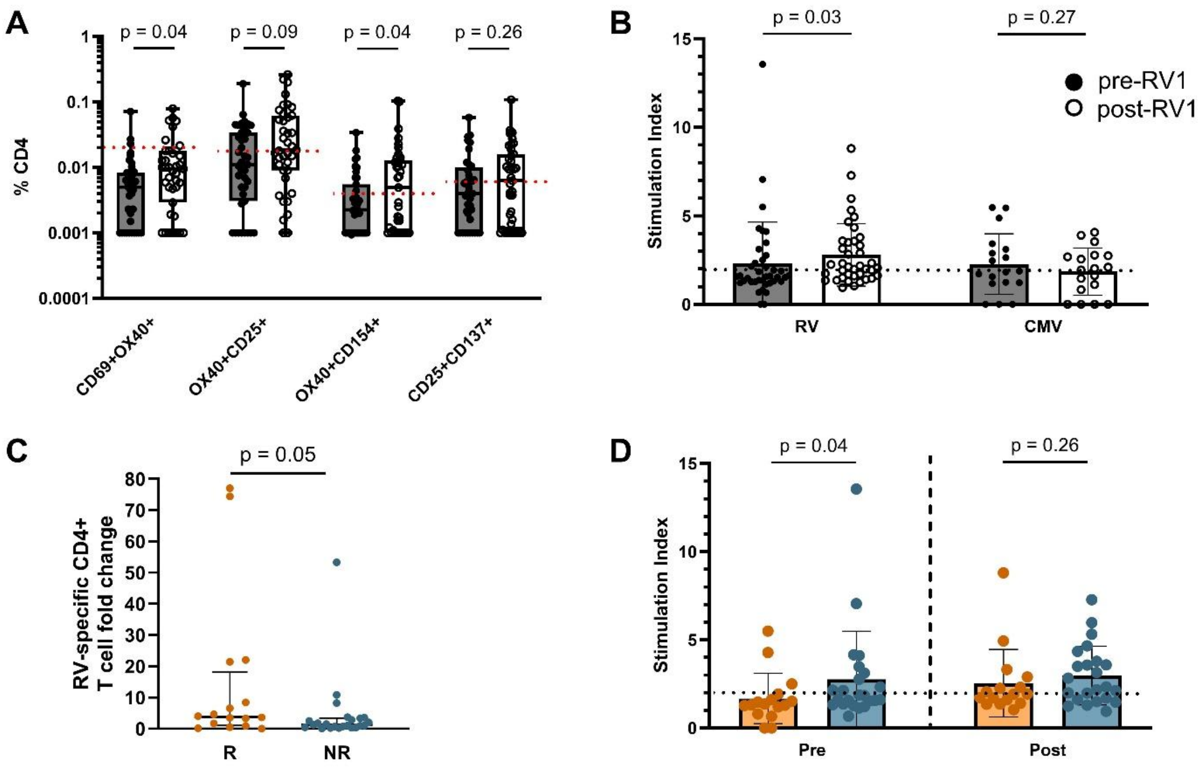

Supplemental Figure 2: Antigen specific CD4+ T cell stimulation indexes pre- and post-RV vaccine. **A.** Paired analysis of DMSO subtracted pre- vs post-vaccine magnitudes of antigen-specific CD4+ T cells identified by each AIM marker combination individually (n = 39). Data are shown with median, quartiles and minimum/maximum values. Horizontal red lines indicate individual LOS defined for each combination (CD69+OX40+ = 0.021, OX40+CD25+ = 0.019, OX40+CD154+ = 0.004, CD25+CD137+ = 0.006). **B.** Paired analysis of stimulation indexes calculated as the magnitude of RV- (n = 39) and CMV-specific (n = 18) CD4+ T cells from Boolean gating divided by the magnitude of the DMSO background. Data are shown with the mean and standard deviation. **C.** Unpaired analysis of fold change of RV specific CD4+ T cells in IgA responders (n = 16) and non-responders (n = 23). Data are shown with the median + IQR. **D.** Unpaired analysis of stimulation indexes split by IgA response status. Data are shown with the mean and standard deviation. Threshold of positivity for SI values (horizontal black dotted line)

is set at ≥2 signifying at least a 2 x difference in magnitude between the antigen-specific and background events Matched pairwise comparisons in A were made by Wilcoxon matched-pairs signed rank tests. Matched pairwise comparisons in B were made by Wilcoxon matched-pairs signed rank tests. Comparisons between IgA response groups in C and D were made by Mann-Whitney tests. Data points represent individual infants. Abbreviations: RV, rotavirus; CMV, cytomegalovirus; IQR, interquartile range; R, RV vaccine serologic responder; NR, RV vaccine serologic non-responder.

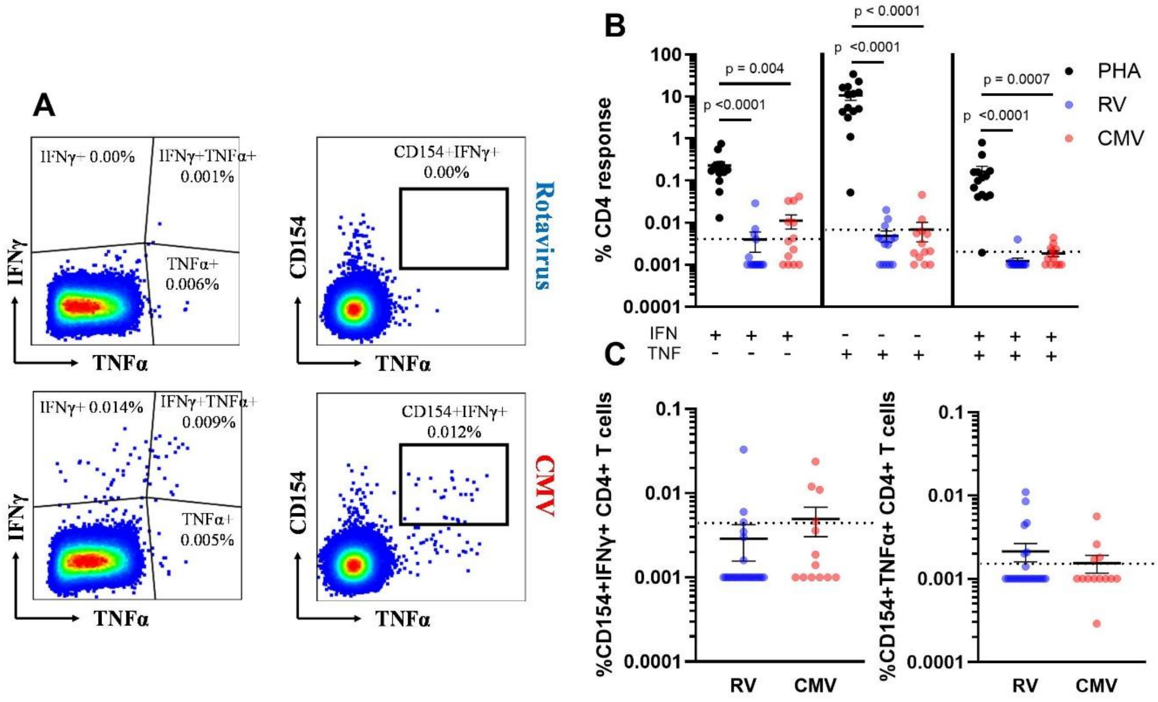

Supplemental Figure 3: Functional responses of RV- and CMV-specific CD4+T cells. A. Representative flow cytometry plot showing the gating strategy for the identification of functional subsets. **B**. Unpaired analysis of functional responses of PHA (black, n = 14), RV (blue, n = 14) and CMV (red, n = 13) stimulated CD4+ T cells. **C.** Unpaired analysis of CD154+ IFNy+ and CD154+ TNFα+ response magnitudes in RV- (n = 24) and CMV-specific (n = 13) CD4+ T cells. Participants were selected based on post vaccine AIM+ stimulation indexes ≥2. Data are shown with the mean and standard error of the mean. Comparisons between RV and CMV phenotypes were made by Mann-Whitney. Comparisons across multiple unmatched groups were made by Kruskal-Wallis tests corrected for multiple comparisons by Dunns post hoc test. Data points represent individual infants. Horizontal lines represent the limit of sensitivity (LOS) for each assay as determined by the median + 2 x standard deviation of a synthetic non-specific myelin oligodendrocyte glycoprotein (MOG) peptide response (B IFNγ = 0.0046, TNFα = 0.006, IFNy/TNFα = 0.0018, C CD154/IFNy = 0.0044, CD154/TNFα = 0.0016).

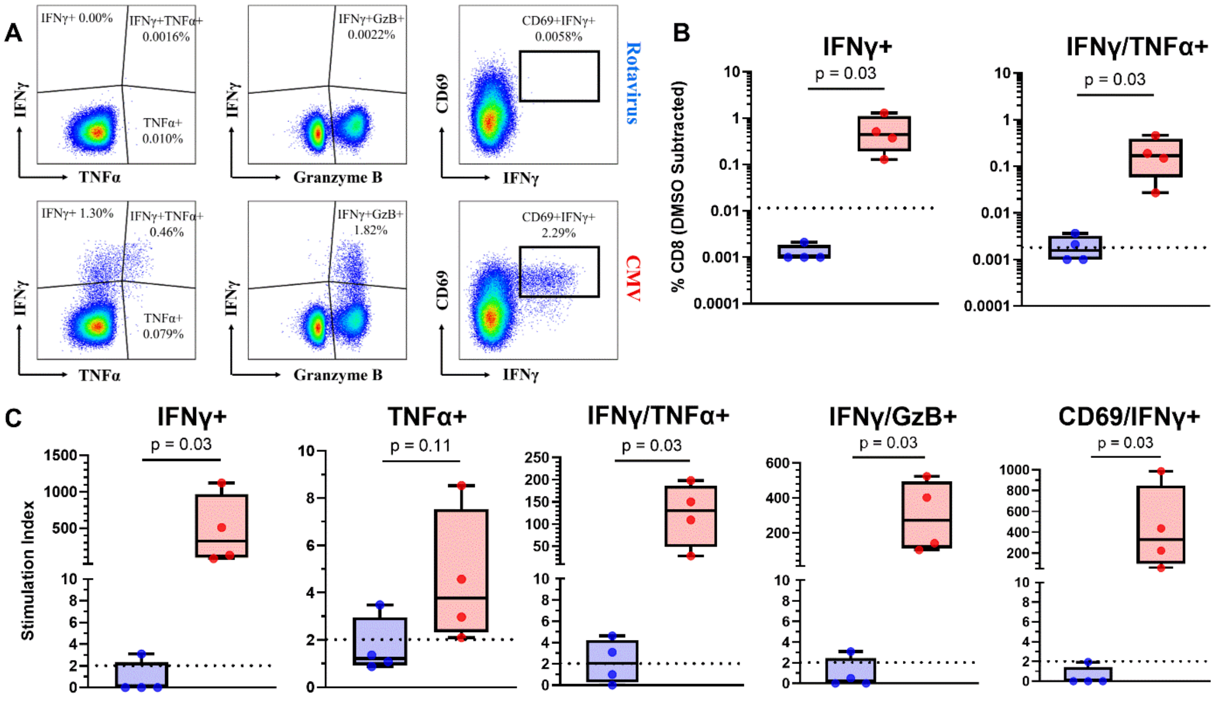

Supplemental Figure 4: Functionality of RV- and CMV-specific CD8+ T cells after 10 hours of stimulation. A. Representative flow cytometry plot showing the gating strategy for the identification of functional subsets after 10 hours of stimulation. **B.** Unpaired analysis of magnitude differences of monofunctional and polyfunctional CD8+ T cells in RV- and CMV-specific T cells (n = 4). Data are shown with median, quartiles and minimum/maximum values **C.** Unpaired analysis of stimulation indexes (SIs) for all functional comparisons between RV- and CMV-specific CD8+ T cells (n = 4). Horizontal lines in B represent the limit of sensitivity (LOS) for each assay as determined by the median + 2 x standard deviation of a synthetic non-specific myelin oligodendrocyte glycoprotein (MOG) peptide response (IFN+ = 0.012, IFN+/TNFa+ = 0.0018). Data are shown with median, quartiles and minimum/maximum values. Data points represent individual infants. Responses are from the same 4 donors. Horizontal dotted lines represent the limit of sensitivity or SI threshold ≥2. Comparisons between RV- and CMV-specific

responses were made via Mann-Whitney tests. Abbreviations: RV, rotavirus; CMV, cytomegalovirus; GzB, granzyme B.

Serum Proteome Variance by Country

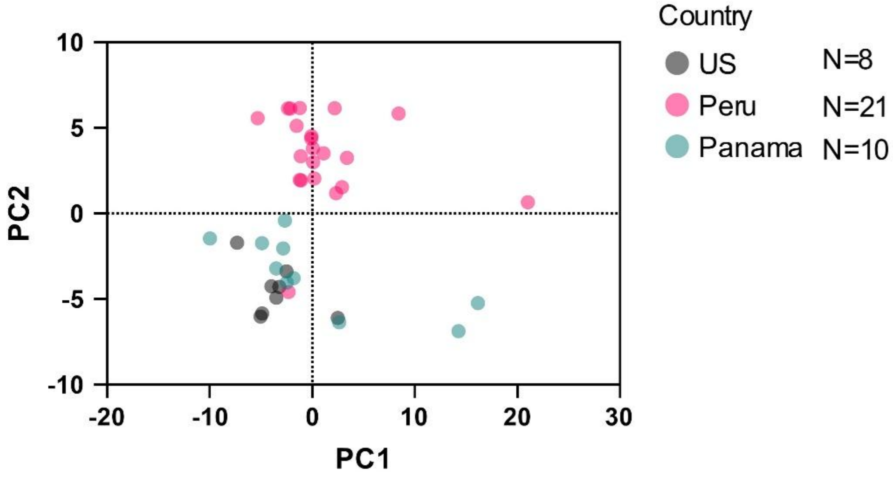

Serum Proteome Variance by IgA Response

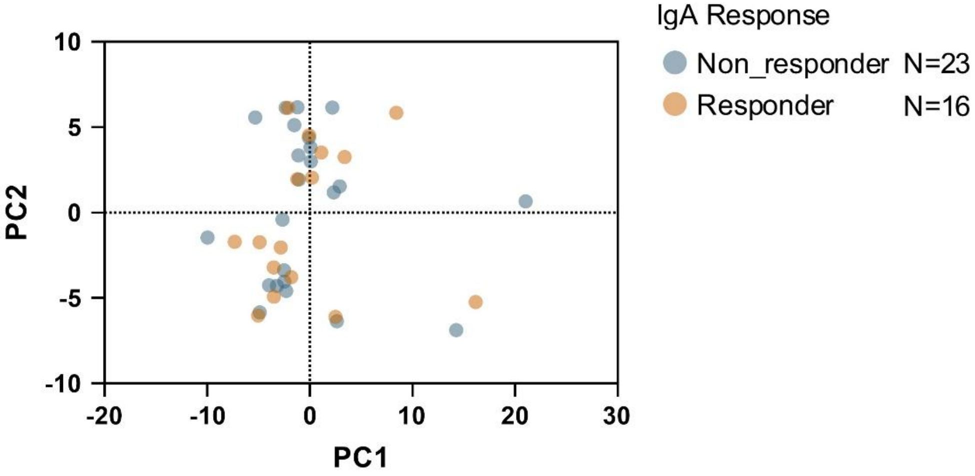

Serum Proteome Variance by T cell Response

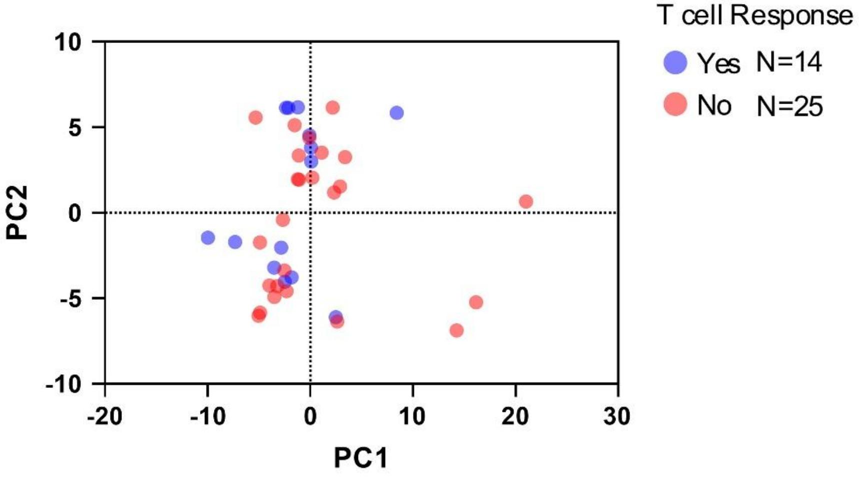

Supplemental Figure 5: Principal component analysis of the 2-month serum proteome. Normalized protein expression data is displayed and was center scaled prior to ordination. Principal components were selected based on their contribution to overall variance with PC1 and PC2 cumulatively explaining 70% of the total variance (PC1 = 45.9%, PC2 = 24.1%). PCA plots are overlayed by **A.** country (US n = 8, Peru n = 21, Panama n = 10), **B.** IgA response status (non-responder n = 23, responder n = 16) and **C** T cell response status (Yes n = 14, No n = 25). Data points represent individual infants.

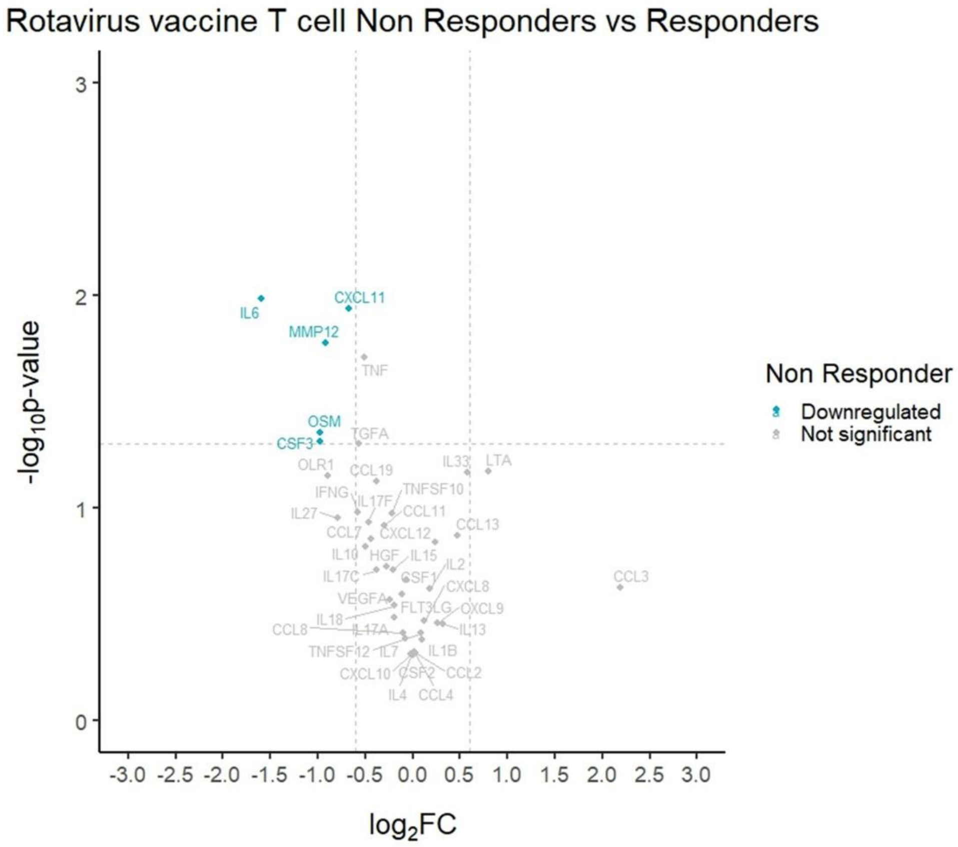
Rotavirus vaccine T cell Non Responders vs Responders
-log10p-value
log2FC
Non Responder
Downregulated
Not significant
IL6
CXCL11
MMP12
TNF
OSM
CSF3
TGFA
CCL3

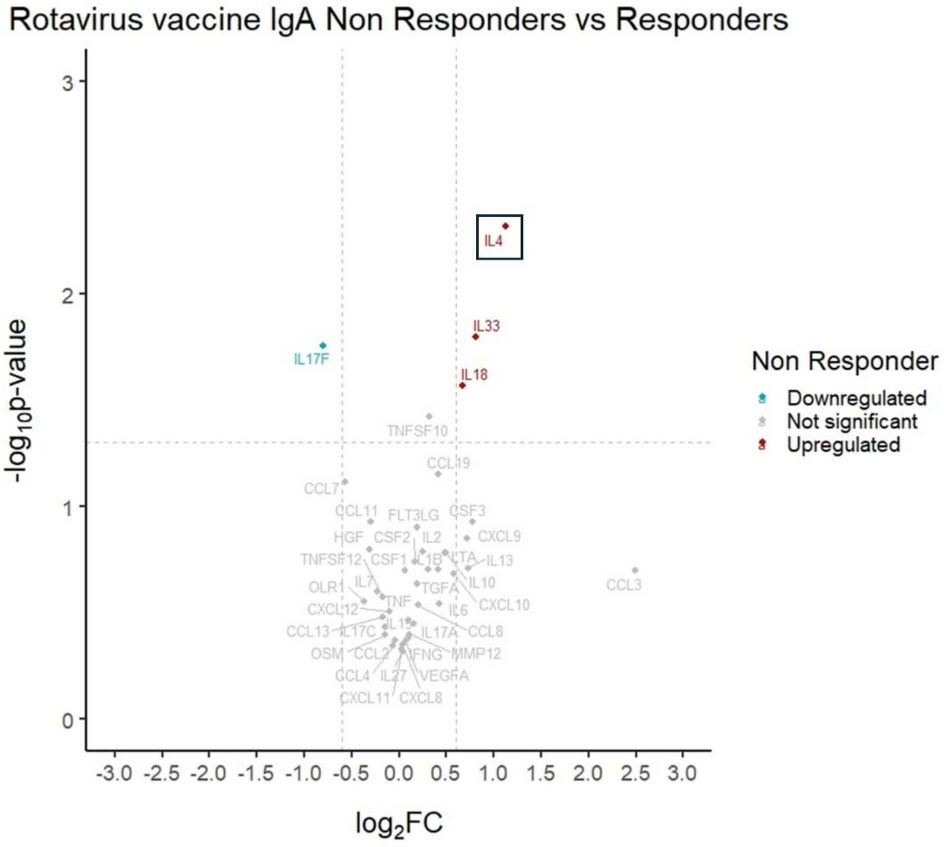
Rotavirus vaccine IgA Non Responders vs Responders
-log10p-value
log2FC
Non Responder
Downregulated
Not significant
Upregulated
IL4
IL33
IL17F
IL18
TNFSF10
CCL3

Supplemental Figure 6: Fold change analysis of serum proteins at 2-months of life by T and IgA response status. Fold changes were calculated by comparing the average T cell non-responder (n = 25) to the average T cell responder (n = 14) and IgA non-responder (n = 23) magnitude to the average responder (n = 16). Fold changes are plotted on the x axis as the log2 of the raw fold change value. p values were calculated for each serum protein and are displayed on the y axis as the -log10 of each unadjusted p value. p values were FDR corrected using the Benjamini-Hochberg method. Proteins with FDR corrected q values <0.1 are highlighted in squares. Horizontal and vertical dotted lines represent the p value cutoff (1.3, corresponding to the -log10 of p = 0.05) and fold change cutoffs (upregulated = log2 of 1.5 = 0.6, downregulated = log2 of 0.5 = -0.6), respectively. Proteins were considered significantly upregulated (red) if the log2 FC was > 0.6 and -1og10 p value >1.3 and downregulated (blue) if the log2 FC was < -0.6 and -1og10 p value >1.3.

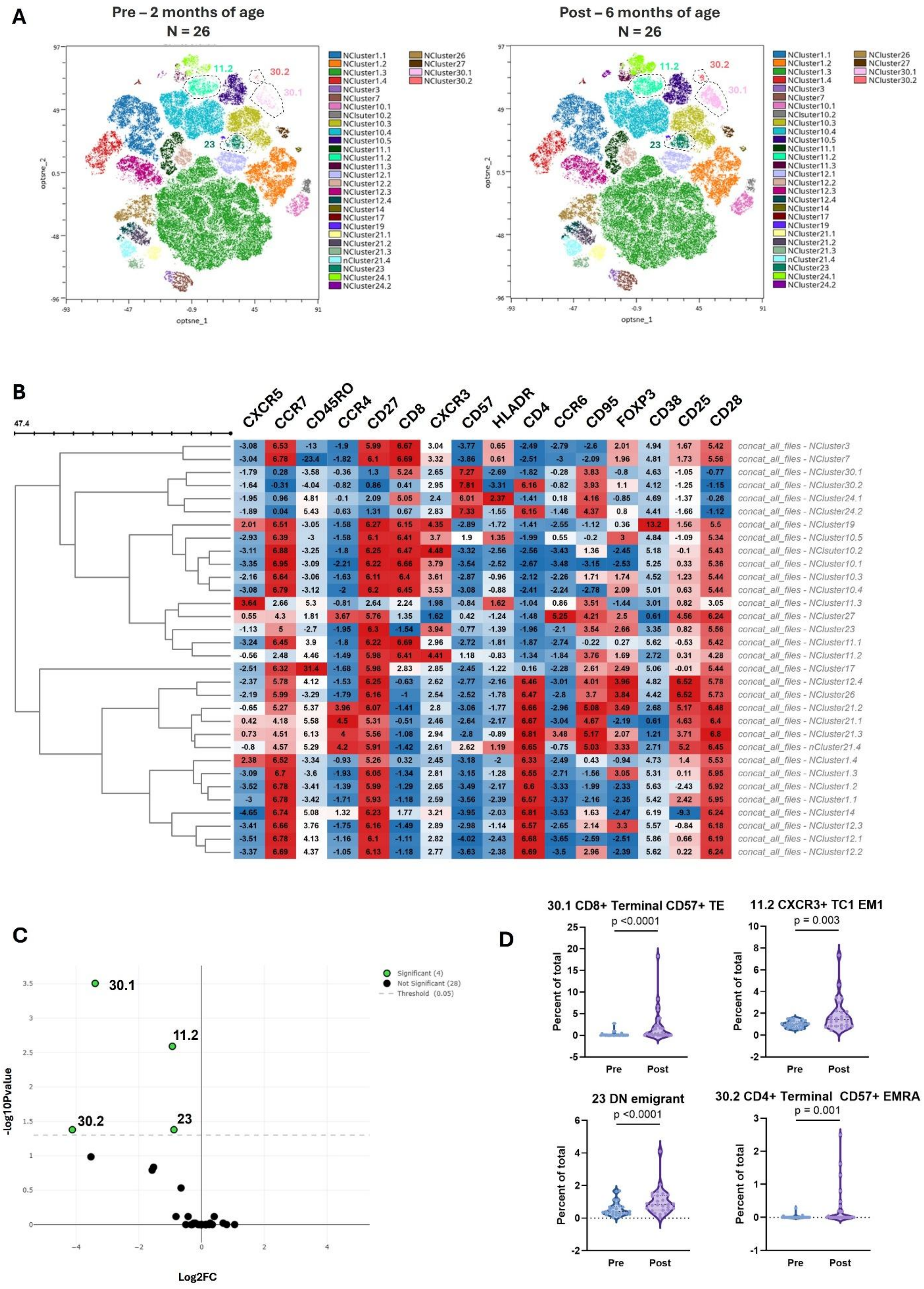

A
Pre – 2 months of age
N = 26
Post – 6 months of age
N = 26
optsne_1
optsne_2
11.2
30.2
30.1
23
NCluster1.1
NCluster1.2
NCluster1.3
NCluster1.4
NCluster3
NCluster7
NCluster10.1
NClsuter10.2
NCluster10.3
NCluster10.4
NCluster10.5
NCluster11.1
NCluster11.2
NCluster11.3
NCluster12.1
NCluster12.2
NCluster12.3
NCluster12.4
NCluster14
NCluster17
NCluster19
NCluster21.1
NCluster21.2
NCluster21.3
nCluster21.4
NCluster23
NCluster24.1
NCluster24.2
NCluster26
NCluster27
NCluster30.1
NCluster30.2
B
47.4
CXCR5
CCR7
CD45RO
CCR4
CD27
CD8
CXCR3
CD57
HLADR
CD4
CCR6
CD95
FOXP3
CD38
CD25
CD28
C
Significant (4)
Not Significant (28)
Threshold (0.05)
30.1
11.2
30.2
23
-log10Pvalue
Log2FC
D
30.1 CD8+ Terminal CD57+ TE
p <0.0001
11.2 CXCR3+ TC1 EM1
p = 0.003
23 DN emigrant
p <0.0001
30.2 CD4+ Terminal CD57+ EMRA
p = 0.001
Percent of total
Pre
Post

Supplemental Figure 7: Bulk T cell phenotyping pre- vs post-vaccine. A. opt-SNE plots with metaclusters overlayed at the pre- and post-vaccine timepoints, n = 26. Statistically relevant clusters are outlined. **B.** Cluster heatmap displaying relative expression of 16 type markers used for FlowSOM analysis. Expression is scaled from negative (blue) to positive (red) with relative values shown. Cluster dendrograms are also shown. **C.** Differential expression analysis by fold change of all 32 metaclusters pre vs post vaccine. Clusters with significant differences in abundance between the two timepoints are displayed in green. Threshold for significance was set at FDR = <0.05. **D.** Identities of the 4 significant clusters found through fold change plotted pairwise pre (light blue) vs post (purple) vaccine, n = 26. Data are shown with the median and quartiles. Data points in D represent individual infants.

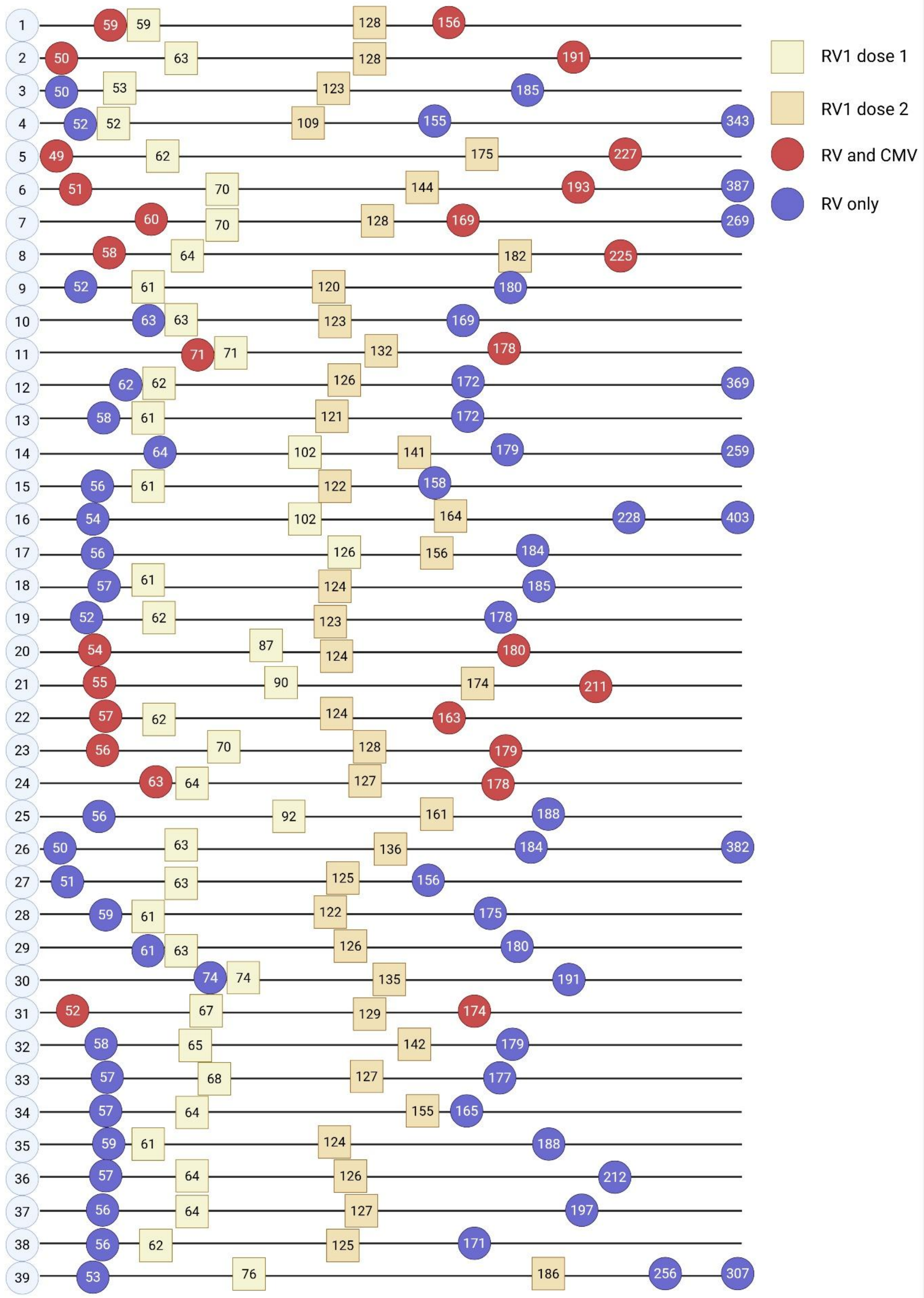

Supplemental Figure 8: Flowchart of participants and T cell assay samples. Participant

number is displayed on the left. Day of life is displayed for all vaccination and blood samples. All participants had RV serology ran for all timepoints. Squares represent vaccination timepoints, circles represent blood draws from which PBMCs for T cell assays were retrieved. Created in BioRender. Nicols, A. (2026) https://BioRender.com/ihwzskc

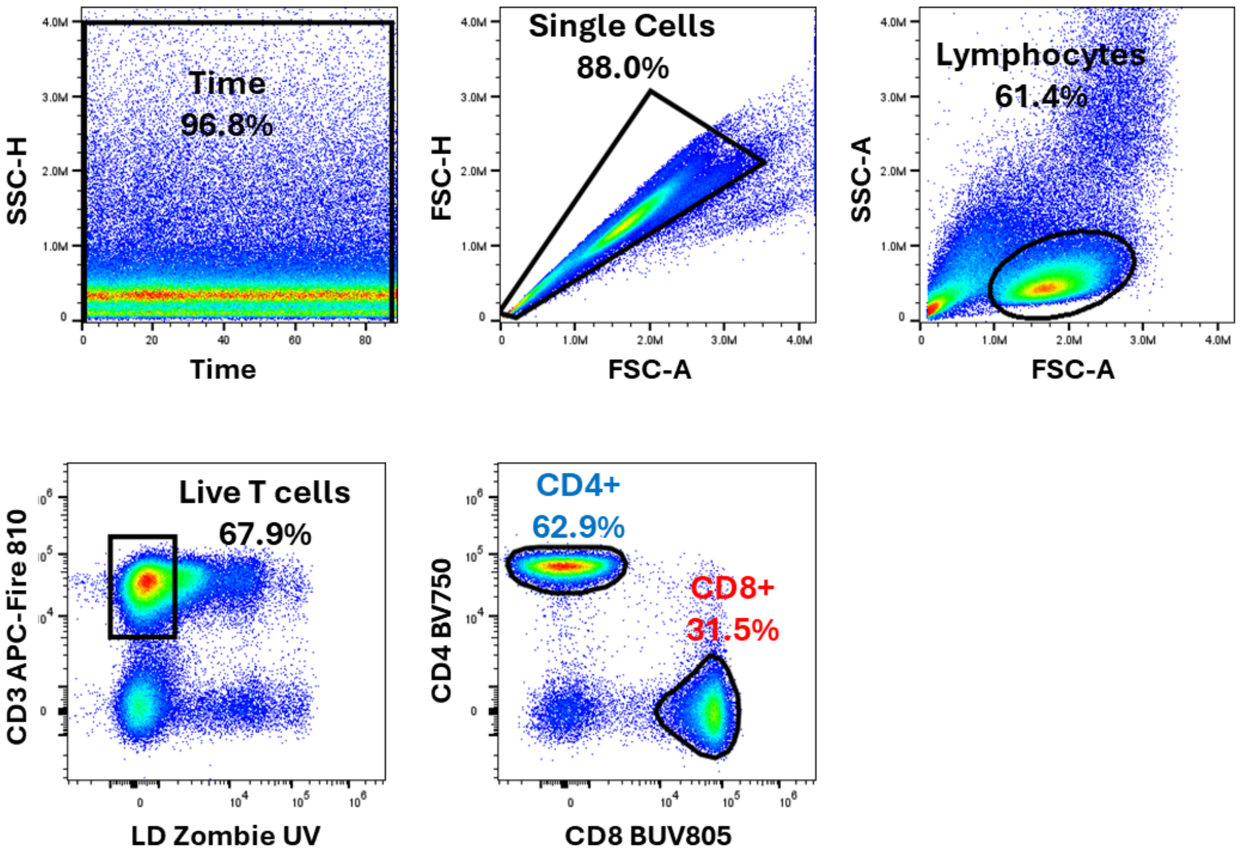

Supplemental Figure 9: Example of sample pre-gating and data cleanup prior to analysis. A time gate was used to gate our regions of aberrant flow at the very beginning and end of a run, single cells were gated based on forward scatter height vs area. Lymphocytes were identified by side scatter area vs forward scatter area gating and live T cells gated based on CD3 positivity and zombie UV negativity. T cells were delineated based on CD4 and CD8 expression prior to downstream analysis.

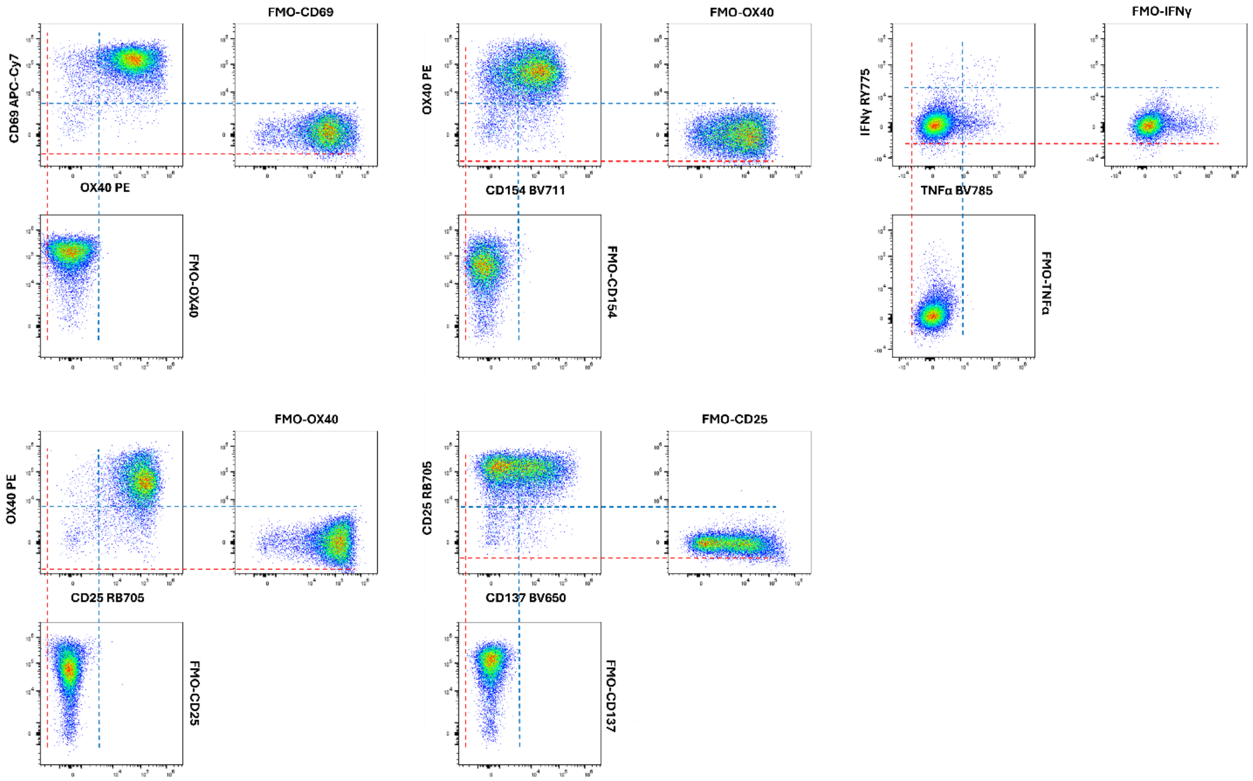

Supplemental Figure 10: AIM+ and functional CD4+ response FMOs. Red dotted lines represent the lower limit of the negative population; blue dotted lines represent the upper limit of the negative population as determined by each respective FMO.

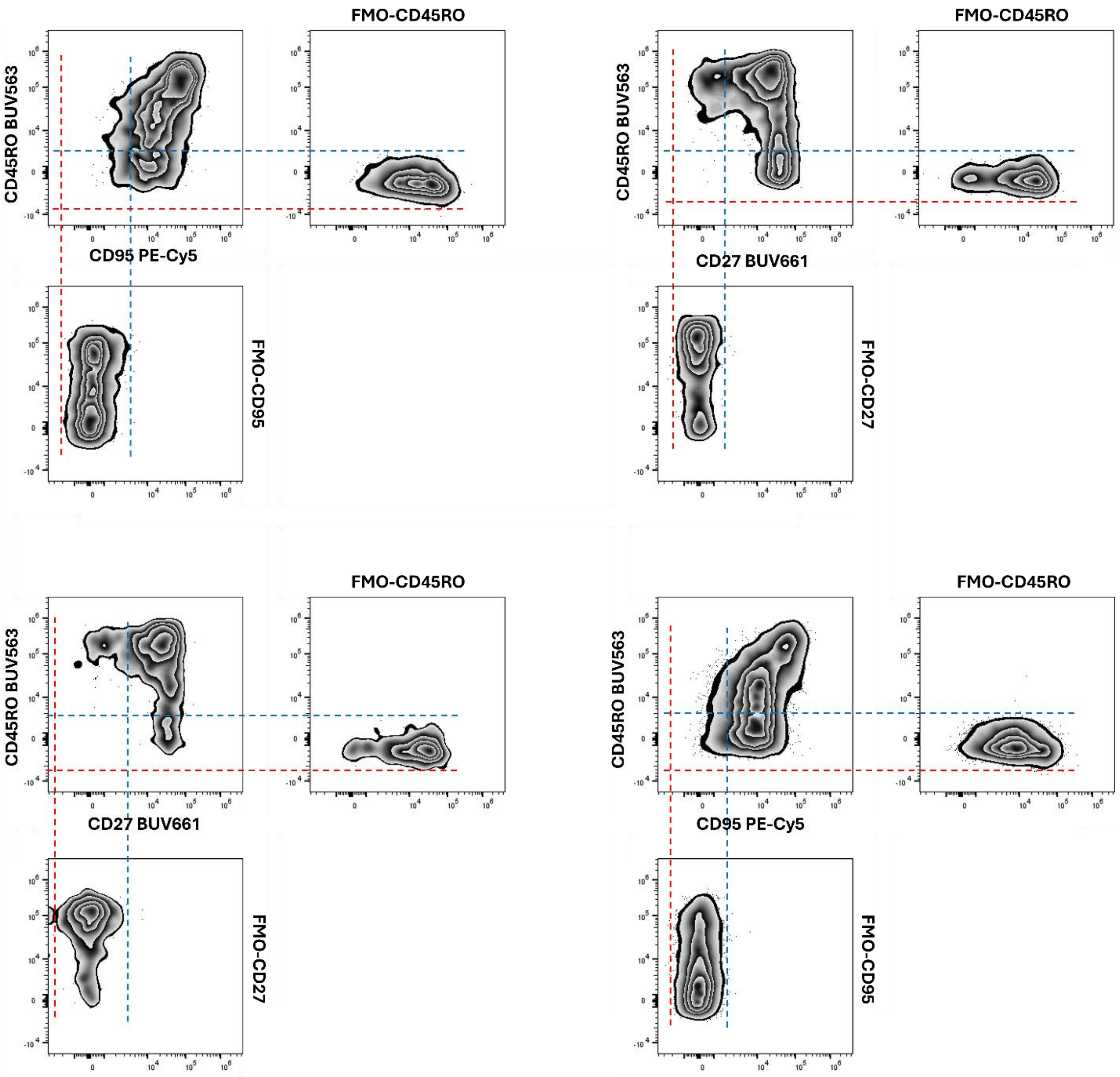

Supplemental Figure 11: Extended memory phenotyping FMOs. Red dotted lines represent the lower limit of the negative population; blue dotted lines represent the upper limit of the negative population as determined by each respective FMO.

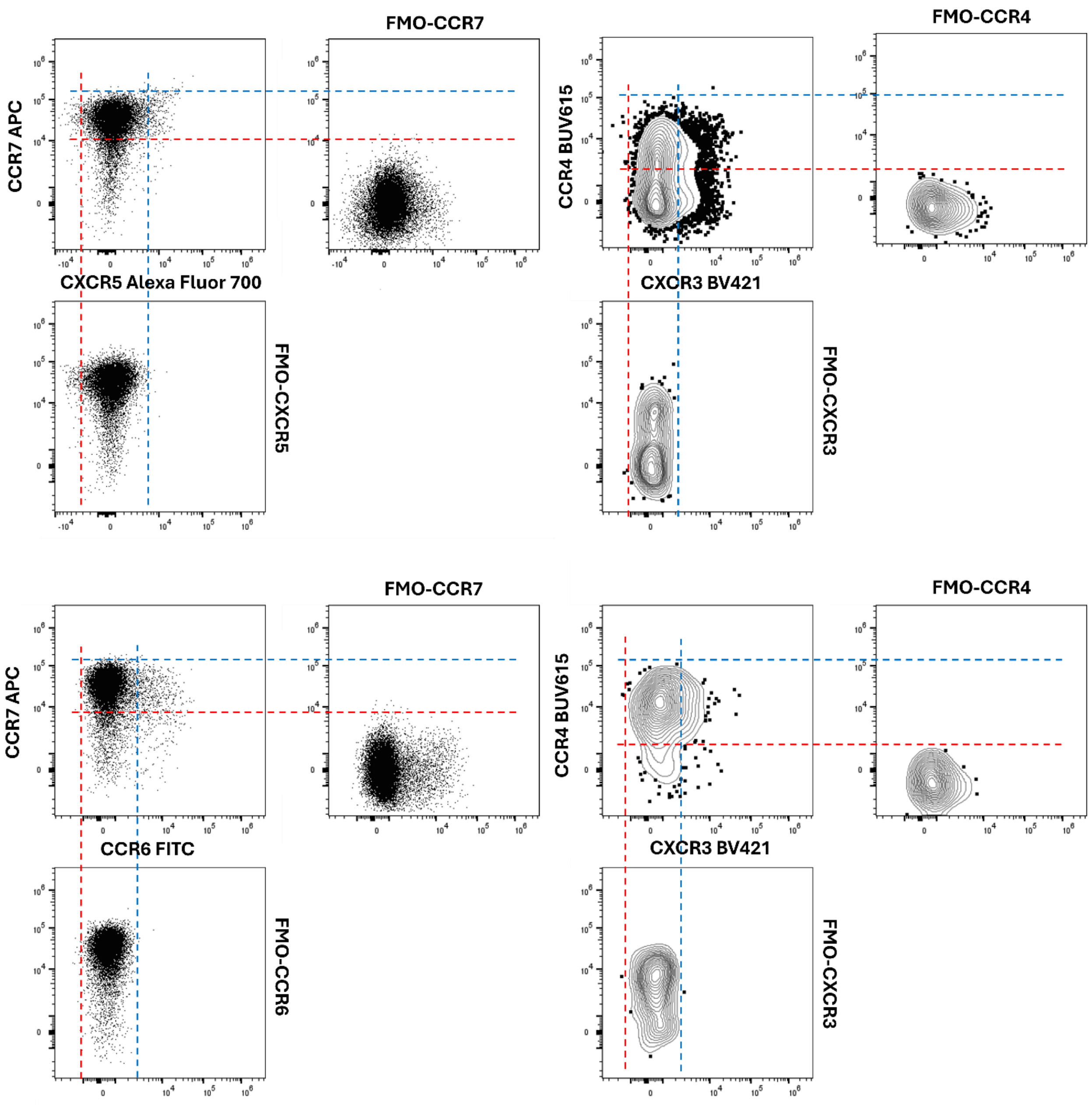

Supplemental Figure 12: Chemokine receptor FMOs. Red dotted lines represent the lower limit of the negative population; blue dotted lines represent the upper limit of the negative population as determined by each respective FMO.

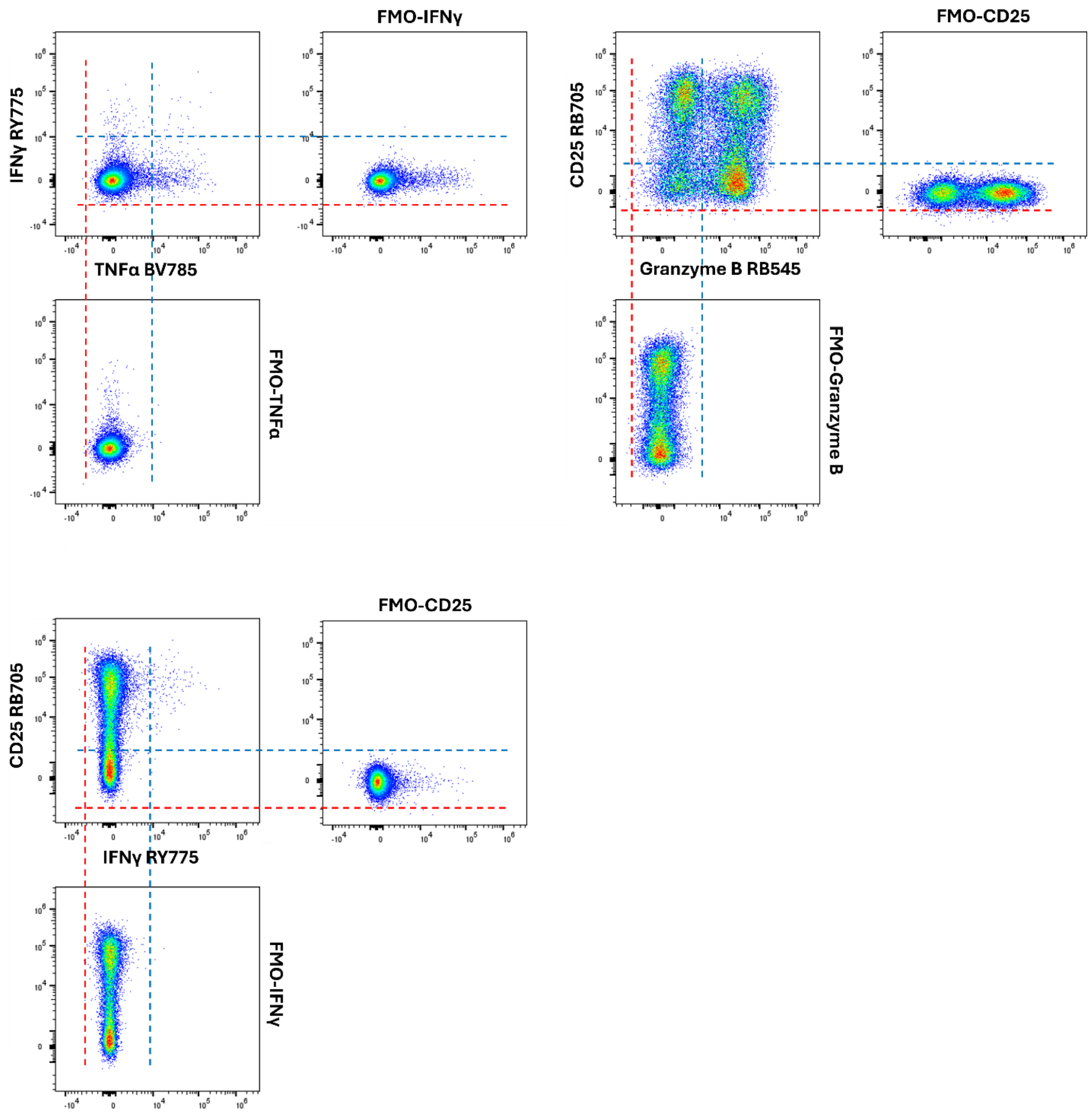

Supplemental Figure 13: Functional CD8+ response FMOs. Red dotted lines represent the lower limit of the negative population; blue dotted lines represent the upper limit of the negative population as determined by each respective FMO.

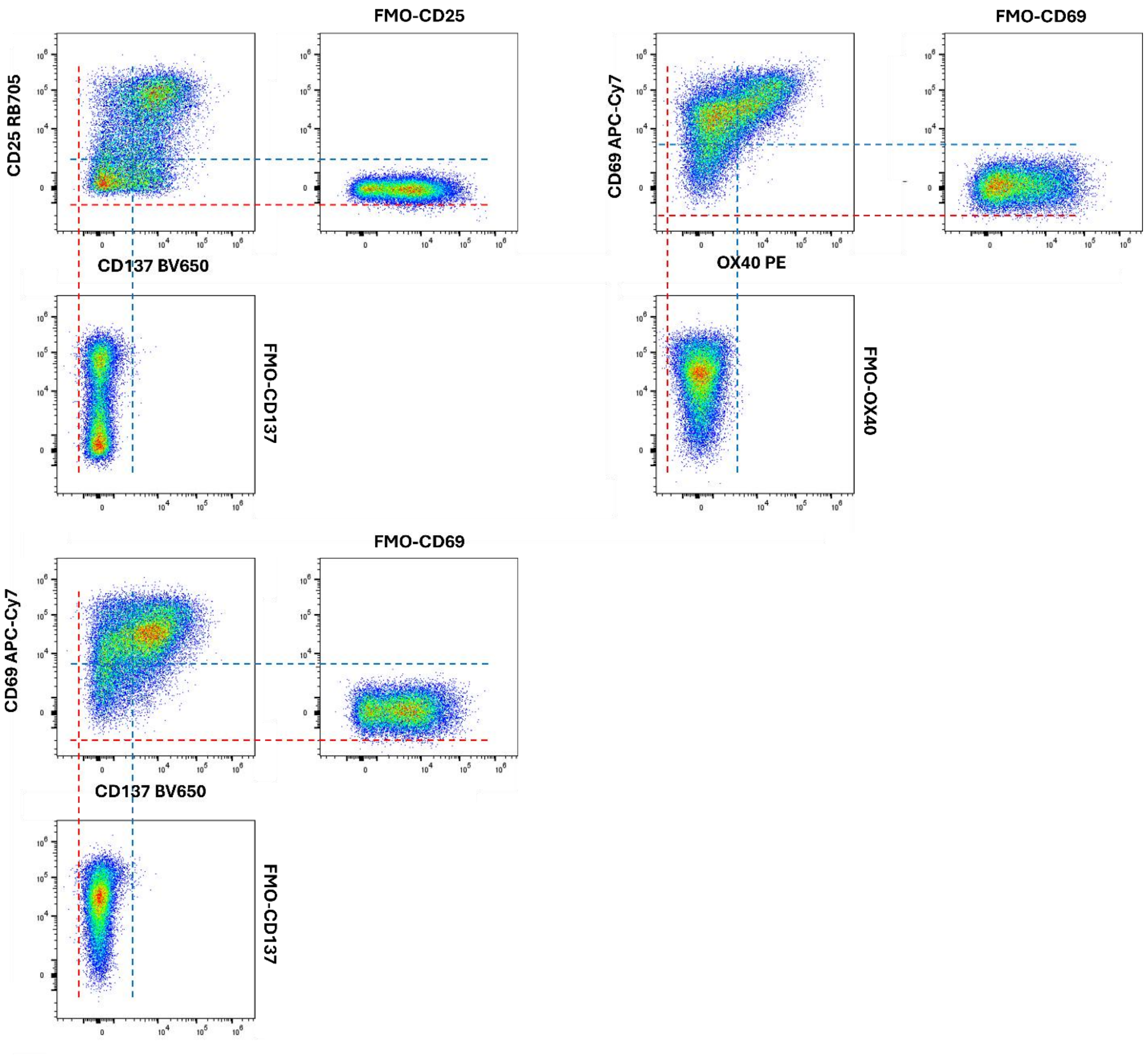

Supplemental Figure 14: AIM+ CD8+ response FMOs. Red dotted lines represent the lower limit of the negative population; blue dotted lines represent the upper limit of the negative population as determined by each respective FMO.
